# The Doctor of Medicine curriculum review at the School of Medicine, Muhimbili University of Health and Allied Sciences, Dar es Salaam, Tanzania: a tracer study report from 2009

**DOI:** 10.1186/s12909-016-0745-7

**Published:** 2016-08-25

**Authors:** Amos Rodger Mwakigonja

**Affiliations:** Department of Pathology, Muhimbili University of Health and Allied Sciences (MUHAS), Dar es Salaam, Tanzania

**Keywords:** Curriculum review, Doctor of medicine, Tracer study, Tanzania

## Abstract

**Background:**

The School of Medicine (SoM) is one among five at Muhimbili University of Health and Allied Sciences (MUHAS). It currently houses eight undergraduate and many post-graduate programmes. The Doctor of Medicine (MD) programme reported herein is the oldest having ten semesters (5 years) followed by a 1 year compulsory rotatory internship at a hospital approved by the Medical Council of Tanganyika (MCT). However, this training was largely knowledge-based and thus the need to shift towards competency-based education (CBE) and full modularization necessitated this study.

**Methods:**

A cross-sectional tracer study of MUHAS MD graduates from SoM who completed training between 2006 and 2008 was conducted using quantitative (structured interviewer-administered questionnaires) as well as qualitative methods [In-depth questionnaire (IDI) and Focus group discussions (FGDs)].

**Results:**

A total of 147 MD graduates were traced and interviewed, representing 29 % of the 510 students who graduated from the SoM between 2006 and 2008. Majority (70.1 %, *n* = 103/147) were males. About 70 % graduated in 2008 and majority (68 %, *n* = 100/147) were doing internship. Majority (60.5 % *n* = 89/147) were based in/near Dar es Salaam at district, regional or referral hospitals. With reasonable concordance, most competencies ranked low except on four aspects. Teaching, System-based Practice and Good Practice had the lowest. Seminars/Tutorials, Laboratory Skills/Practicals, Theatre Skills, Outpatients clinics, Family Case Studies, Visits/Excursions and Self Reflection were rated less useful teaching methods compared to Lectures, Teaching Ward Rounds, Elective Studies, Field Work, Presentations, Continuous Assessments Tests, Final Examinations, Short Answers, Clinical/Practical Examinations. ICT and Library facilities were not considered to meet the students learning needs and Clinical Logbooks also ranked low. Teachers were generally ranked less favorably including in professional role-modelling and accessibility outside scheduled teaching sessions.

**Conclusions:**

This tracer study results allowed subsequent curriculum review and the introduction of full modularization and competency-based learning at MUHAS. It is envisioned that these tracer study findings will improve teaching, learning and inform next curriculum review at MUHAS leading to increased output of appropriately trained health professionals to fill the big gap in human resources for health (HRH) in Tanzania. The revised curricula are also being processed through TCU for accreditation as required.

**Electronic supplementary material:**

The online version of this article (doi:10.1186/s12909-016-0745-7) contains supplementary material, which is available to authorized users.

## Background

Curricula for academic programs need to be reviewed from time to time in order to ensure that they are current with respect to socio-economic and demographic changes at national and global levels, as well as changes in community needs and technological advances [1]. In the case of curricula for health sciences, reviews also need to take into consideration the changing pattern of disease occurrence and the increasing demands for health care. Therefore, the University recommends review of academic curricula after every 5 years. However, curriculum revision should always be informed by findings from tracer studies as well as being guided by assessment for competencies and skills including their reflections on the curriculum they went through and how much they find it useful/not useful during their post-graduation practice [1, 2]. The revised curricula should also be processed through the Tanzania Commission for Universities (TCU) for accreditation as required. It is in this light that the School of Medicine as well as other schools at MUHAS have conducted two tracer studies since the commencement of the semesterized programmes about 7 years ago [3]. In these studies, medical graduates, their co-workers, employers as well as their end-users (patients) were interviewed while selective graduates also had in-depth questionnaires and focus group discussions. Herein we report the results of the second tracer study. The aim was to evaluate how much graduates displayed those competencies and skills and whether they performed better or worse compared to graduates from other medical schools [3].

### Setting of the School of Medicine

The School of Medicine started as the Dar es Salaam School of Medicine in 1963 with initial intake of eight students. In 1968, the school was upgraded to a Faculty of Medicine of the Dar es Salaam University College, which was a constituent College of the University of East Africa [4].

In 1970, the University of East Africa ceased to exist and the Dar es Salaam University College became the University of Dar es Salaam, whereby the Faculty of Medicine became one of its units. In 1977, following the creation of Muhimbili Medical Centre (MMC), the Faculty of Medicine administratively merged with Muhimbili Hospital. Subsequently, in 1991, the Faculty of Medicine was upgraded to a College and its name changed to Muhimbili University College of Health Sciences (MUCHS), consisting of four Faculties (Medicine, Pharmacy, Dentistry and Nursing) and five institutes (Public Health, Traditional Medicine, Primary Health Care and Continuing Medical Education, Development Studies and Allied Health Sciences). In 2003, after MMC ceased to exist, MUCHS and Muhimbili National Hospital (MNH) became autonomous and the Faculty of Medicine was changed to the School of Medicine (SoM) [4]. Beginning January 2007, the SoM is one of the units under the Muhimbili University of Health and Allied Sciences (MUHAS). This followed the upgrading of MUCHS into MUHAS following the signing of the charter establishing the University on 28th March 2007 by H.E. the President of the United Republic of Tanzania [4].

### The former and current tracer studies

The first tracer study was conducted in 2003 by the then Muhimbili University College of Health Sciences (MUCHS) thus there was a need to revise the existing curriculum in 2009. This activity was preceded by a change of the former term system to a semesterized teaching system. The aim of the first Tracer Study was to obtain baseline information from the former graduates, their employers and end users for the purpose of guiding the curriculum review process for the different academic programs and also to serve as a point of reference for future follow-up tracer studies. Specifically the first Tracer Study aimed to establish the needs of employers of MUCHS graduates; determine the market demand for the MUCHS graduates; obtain information on the adequacy of the existing programs at MUCHS; establish the expectations of the end-users regarding the roles of MUCHS graduates and the level of acceptability of the MUCHS graduates to the community they serve.

The current tracer study was the second one that was conducted in order to generate information that would inform revision of the curriculum [2]. The overall goal was to obtain important information for developing competency-based curricula that are relevant to the national socio-economic development; respond to market demands and stakeholders needs; and conform to MUHAS vision and educational standards. It is envisioned that this would in turn improve teaching and learning and lead to increased output of appropriately trained health professionals to fill the big gap in human resources for health in the country [3, 5].

The current tracer study was included in the Academic Learning Project which was funded by the Bill and Melinda Gates Foundation through the MUHAS-UCSF Partnership under the Directorate of Continuing Education and Professional Development (DCEPD) at MUHAS [1, 6, 7].

### Purpose of the tracer study and development of competency-based MD curriculum

The current tracer study report has covered MD graduates only because BMLS and BSc. RTT programmes were still new at the time of revision and did not yet have graduates to be traced. In this regard, recent medical graduates from SoM who had undergone training through the semesterized MD programme, where assessed as to how much they felt they possessed competencies and skills including the MUHAS core competencies as well as those specific for the MD degree; and how much they felt enabled/empowered or disadvantaged by the curriculum they went through [3]. The overall goal was to develop competency-based curricula,that are relevant to the national socio-economic development; respond to market demands and stakeholders needs; and conform to MUHAS vision and educational standards. The findings of the index tracer study directly informed and guided the ongoing curriculum review exercise including the introduction of MUHAS core as well as MD-specific competencies and skills; repackaging of the curriculum, full modularization, increased inter-professional interactions and teamwork as well as other improvements.

## Methods

### Study design

This was a cross-sectional tracer study of MUHAS MD graduates from SoM who completed training between 2006 and 2008 was conducted using quantitative (structured interviewer-administered questionnaires) as well as qualitative methods [In-depth questionnaire (IDI) and Focus group discussions (FGDs)].

### Study area

The study was based at the School of Medicine (SoM) which is one among five at Muhimbili University of Health and Allied Sciences (MUHAS), Dar es Salaam, Tanzania. It currently houses eight undergraduate and many post-graduate programmes (Additional file 1 attached).

The Tracer Study tools were designed based on the information obtained from the gap analysis exercise that was carried out for each course by representatives of all Schools and Institutes.

### Sampling and inclusion/exclusion criteria

In August 2009, teams of MUHAS academic staff and students traveled to Ilala, Kinondoni, Temeke, Mkuranga, Kisarawe, Bagamoyo, Rufiji, Mwanza, Mbeya, Arusha, Moshi, Tanga and Dodoma to administer questionnaires and interviews. These areas represented Dar es Salaam and upcountry regions and also were chosen for logistical reasons including proximity and presence of major tertiary hospitals were many graduates could be found.

Medical students who had graduated between 2006 and 2008 and were located at their places of employment (hospitals, clinics, dispensaries, government agencies and other places) were included, as well as their co-workers, supervisors and employers. In some cases their clients/patients were also interviewed. Graduates before 2006 were not included since they did not use the existing semesterized curriculum. Non-MD graduates were also excluded from this study.

### Quantitative methods

The topics of the questionnaires covered: 1) the demographics of those interviewed; 2) assessment of the competencies of MUHAS graduates by the graduates and by their employers; 3) assessment by the graduates of the relevance and organization of the courses at MUHAS; and 4) assessment by the graduates of the learning/teaching environment (Additional file 2).

The competencies used in the questionnaires were grouped as: a) general competencies expected of all MUHAS graduates; b) specific professional competencies expected of the graduates from a school/institute; and c) specific clinical/practical competencies expected of the graduates from a school/institute (Additional file 3).

Survey data were entered into the database program, WAMP, and then transferred to SPSS for analysis.

All opinions were scored using a Likert scale ranging from 1 (strongly disagree) to 5 (strongly agree). For each item scored using the Likert scale, mean and standard deviation scores were calculated as well as the percentage of respondents who agreed with the statement (that is responses of either “strongly agree” or “agree”). Thus, figures in cells (Additional file 4) are the percentages agreeing, mean (SD) of Likert Scale from 1 to 5. A mean reading of ≥4 is defined as high while that <4 is low.

### Qualitative methods

#### In-depth questionnaire (IDI)

During this tracer study, the School of Medicine also conducted qualitative research including in-depth interviews (IDI) as well as focused group discussions (FGD).

Recent SoM graduates were surveyed about their experiences with the MUHAS medical curriculum. IDI responses were solicited in regards to specific courses of their choosing. The main areas that students were asked to comment on were the strengths of the course, the weaknesses of the course and suggestions for improvements to the course.

Several overarching themes emerged from these responses; including references to professors, practical experience, over enrolment, facilities and educational materials, curricular content and finally duration of courses. Each main theme will be defined and discussed with references to strengths, weaknesses and necessary improvements.

The preliminary analyses were prepared by members of the Academic Learning Project (ALP). Further analyses were conducted by the ALP Metrics Team after findings were shared with the Schools and Institute.

#### Focus group discussions (FGDs)

A series of focus groups were conducted in the summer of 2009 with recent graduates from the Muhimbili University of Health and Allied Sciences (MUHAS) and these were conducted in conjunction with University of California, San Francisco (UCSF). These focus groups were made up of six to eight recent graduates who had finished their programme of study in the spring of 2009. Students were grouped according to the school from which they graduated. There were six groups representing the Medical, Dentistry, Nursing, Pharmacy Schools and the Allied Health Science and Environmental Health programs.

The discussions explored graduates perceptions of the curriculum and possible improvements. Discussion focused on over enrolment, facilities, supplies and faculty, preparation for practical work and fieldwork experiences. Students provided very specific recommendations for each health science school, which is included in a section of this report.

Discussions were semi-structured and included prompt questions soliciting opinions on the challenges of the curriculum and how students propose to improve them Focus group discussions were recorded and transcribed. Data was then coded and analyzed according to major themes.

In order to provide information for the curriculum revision, this report focuses mostly on constructive criticism directed at areas in need of improvement whereas in the focus group discussions focused both on praise and criticism. Major themes of discussion included facilities and supplies, over enrolment, educational time frame, theoretical preparation for practical work, experiential learning and fieldwork, communication, course load, skills and faculty. These themes are discussed below, followed by specific improvements that were proposed in each school.

##### Participants

The student focus groups were held in July of 2009 with 35 students who had recently completed the degree in medicine. The focus group intended to seek information from these most recent graduates about their perceptions regarding the training that they received at MUHAS and professional competency. The 2009 graduates were our target focus group participants as they were not included in the Tracer study having not yet assumed professional/ internship positions.

##### Questionnaire design

The focus group questions were designed by a team of MUHAS and UCSF faculty and students. The questions/prompts intended to illicit responses that would inform curriculum revisions. The questions explored the graduates’ perceptions around how competency is built and assessed and asked for specific feedback on programs of study. In this respect, the questions used were derived from the themes of questions in the Tracer study (Additional file 2).

##### Data analysis

The focus group discussions were tape recorded and transcribed. Translations were done from Swahili to English where needed. Transcripts were coded thematically. Results were analyzed according to theme across schools. These results use cross-cutting themes to report on the combined data from all schools. Additionally, student recommendations for specific programs were compiled according to school.

## Tracer study limitations

1. Financial constraints limited coverage and duration of the study thus could not allow the tracing of graduates who were working in remote areas/or temporarily away from their work places
2. The tracer study covered mostly centers within urban/semi-urban health care facilities
3. Some few graduates were available but did not participate
4. The questionnaire coding system did not allow the tallying of responses from co-workers, supervisor and employer to the graduate to allow direct feedback.

## Results

### Quantitative studies results

#### General demographics and geographic coverage of MD graduates

**Fig. 1:**
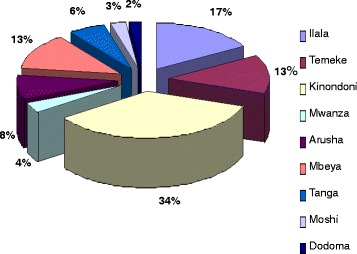
Pie Chart showing the distribution of interviewed graduates by supervisor by site (district of working station). Majority were working in/near Dar es Salaam

**Fig. 2:**
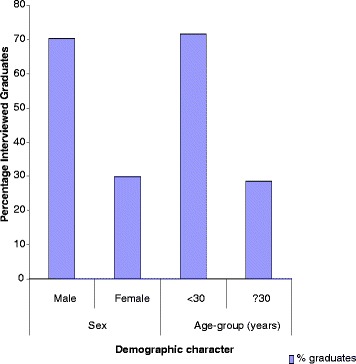
Bar Chart showing the demographic characteristics of interviewed graduates. Majority of MUHAS graduates during the study period were males below 30 years of age

A total of 147 MUHAS MD Graduates were traced and interviewed, representing 29 % of the 510 students who graduated from the School of Medicine between 2006 and 2008 (Table 1). Seventy percent of those interviewed had graduated in 2008, representing 51 % of those who had graduated in that year. The distribution by district of employment is shown in Table 2. Majority (60.5 %, *n* = 89/147) were based in or near Dar es Salaam (Fig. 1). Only 3 graduates were missing during the interviews and all were from 2009. Majority of graduates during the study period were males below 30 years of age although data on age for some few (5.4 %, *n* = 8/147) participants was missing (Fig. 2, Table 3). However, data on sex was available for all interviewed graduates. The distribution of MD graduates according to the type of employment and organization where they are currently employed is shown in Table 1.

**Table 1:** Distribution of MUHAS MD graduates by employing organization

Employing organization	Ministry or district health administration	37	25.2
	National hospital	14	9.5
	Regional or referral hospital	34	23.1
	Private or religious hospital	11	7.5
	University or training institution	9	6.1
	International non-governmental organization or agency	1	0.7
	Other	17	11.6
	Missing	24	16.3
Total		147	100.0

*NB* Majority of MUHAS graduates during the study period were working in public hospitals in either referral, regional or district health administrations

**Table 2:** MD graduates interviewed for the 2009 Tracer Study

		Total number who graduated	Number interviewed	Number interviewed as a % of those who graduated	Percentage interviewed by year of graduation
Graduation year	2006	134	12	9.0	8.2
	2007	175	28	16	19.0
	2008	201	103	51.2	70.1
	2009		1	–	0.7
	Missing		3	–	2.0
Total		510	147	29.2	100.0

**Table 3:** Demographics and employment status of the interviewed graduates

		Number	%
Sex	Male	103	70.1
	Female	44	29.9
	Missing	0	0.0
Total		147	100.0
Age in years	25	6	4.1
	26	13	8.8
	27	17	11.6
	28	41	27.9
	29	28	19.0
	30	15	10.2
	31	6	4.1
	32	5	3.4
	34	2	1.4
	35	2	1.4
	38	1	.7
	39	3	2.0
	Missing	8	5.4
Total		147	100.0
Employment status	Intern	100	68.0
	Full time	33	22.4
	Part time	1	.7
	Contract	13	8.8
	Missing	0	0.0
Total		147	100.0

#### General observations

Majority (60.5 %) of graduates from the 2008 year group were based around the Dar es Salaam region. Majority of these graduates were male and working as interns (housemen). Most competencies ranked low including teaching skills, health care system and good practice which had the lowest and respondents seemed generally in agreement were rating was low (Table 4).

**Table 4:** Competencies common to all MUHAS graduates

Competence Figures in cells are the percentages agreeing, mean (SD) of Likert Scale from 1 to 5, where 1 = strongly disagree and 5 = strongly agree	Graduate *n* = 147	Supervisor *n* = 48	Supervisor *n* = 48
	I was “trained to”	“Professionals are expected to”	“Graduate is able to”
B1: Relationships with patients			
Establish constructive relationships and communicate effectively with patients, clients and/or communities in order to address their needs and preferences	84.9 %	95.6 %	80.0 %
	4.29 (0.90)	4.71 (0.73)	3.98 (0.89)
Provide service to individuals and groups that is appropriate to their different backgrounds	80.6 %	95.5 %	72.7 %
	4.17 (1.02)	4.70 (0.73)	3.82 (1.10)
Communicate health issues and polices effectively to the public	69.2 %	95.3 %	72.1 %
	3.92 (1.08)	4.74 (0.73)	3.79 (1.08)
B2: Relationships with colleagues			
Listen to and take advice from colleagues	84.2 %	97.7 %	68.3 %
	4.34 (1.06)	4.82 (0.66)	3.68 (1.16)
Motivate colleagues	72.6 %	97.6 %	56.8 %
	3.97 (1.11)	4.68 (0.72)	3.55 (1.13)
Contribute effectively to team work	82.2 %	97.7 %	65.9 %
	4.27 0 (.90)	4.88 (0.62)	3.86 (0.95)
Work effectively with other health professionals	84.7 %	97.7 %	75.0 %
	4.30 (1.00)	4.82 (0.66)	4.07 (.90)
B3: Teaching			
Prepare and deliver effective health promotion messages to educate communities	67.8 %	95.3 %	52.3 %
	3.84 (1.08)	4.58 (0.76)	3.50 (1.02)
Teach a course for health professionals or students	65.3 %	86.4 %	46.5 %
	3.78 (1.26)	4.50 (0.93)	3.51 (0.93)
B4: Good practice			
Systematically evaluate one’s own performance and practice	64.8 %	95.5 %	34.1 %
	3.69 (1.11)	4.57 (0.76)	3.27 (1.06)
Regularly seek information necessary to improve professional practice (life-long learning)	75.3 %	97.7 %	59.1 %
	4.05 (1.10)	4.77 (0.68)	3.66 (1.14)
Apply evidence-based decision making	74.3 %	97.6 %	59.5 %
	4.03 (1.04)	4.67 (0.72)	3.57 (1.00)
Participate in applied research activities	67.8 %	90.2 %	50.0 %
	3.88 (1.16)	4.51 (0.75)	3.41 (1.23)
Use information technology to optimize learning	50.7 %	97.6 %	55.0 %
	3.48 (1.20)	4.71 (0.60)	3.65 (1.08)
Show leadership and managerial skills	56.6 %	95.3 %	47.7 %
	3.66 (1.15)	4.65 (0.65)	3.30 (1.17)
B5: Health care systems			
Show knowledge of how the health care system functions (structures, policies, regulations, standards and guidelines)	60.0 %	90.7 %	44.4 %
	3.80 (0.94)	4.56 (0.73)	3.44 (1.08)
Work effectively in various health care delivery settings and systems (hospitals, government, ministries, NGO’s, communities, industry)	63.2 %	97.7 %	64.4 %
	3.83 (1.00)	4.72 (0.59)	3.82 (1.01)
Coordinate and implement health service delivery and health interventions within the health care system	66.9 %	97.7 %	53.3v
	3.83 (1.048)	4.60 (0.62)	3.47 (0.94)
Incorporate considerations of cost effectiveness into health service delivery	63.4 %	88.4 %	52.3 %
	3.83 (1.02)	4.51 (0.77)	3.45 (1.00)
Incorporate considerations of patient cost burden into health service delivery.	67.6 %	90.9 %	50.0 %
	3.83 (1.04)	4.50 (0.73)	3.39 (1.06)
Promote quality care in health systems through audits, accreditations, and/or evaluations	48.3 %	93.0 %	46.5 %
	3.43 (1.22)	4.56 (0.70)	3.33 (1.15)
Identify system challenges and implement potential solutions	55.2 %	94.9 %	46.5 %
	3.59 (1.18)	4.56 (0.79)	3.44 (1.16)
Maintain ethical standards (confidentiality, informed consent, avoid practice errors, avoid conflicts of interest)	94.5 %	97.7 %	73.9 %
	4.59 (.70)	4.77 (0.68)	3.87 (1.24)
Apply entrepreneurial skills for advancement of practice and the profession	51.0 %	88.1 %	51.1 %
	3.46 (1.28)	4.38 (0.901)	3.47 (1.16)
Show sensitivity and responsiveness to diversity (culture, age, socioeconomic status, gender, religion, and disability)	70.3 %	97.7 %	63.6 %
	4.03 (1.03)	4.65 (.72)	3.73 (1.17)
Show respect, compassion, and integrity while interacting with patients, clients, communities and health professionals	88.3 %	97.7 %	68.9 %
	4.44 (0.86)	4.74 (0.69)	3.80 (1.14)
Advocate and implement fair distribution of health care resources in Tanzania	64.3 %	90.7 %	51.2 %
	3.85 (1.14)	4.63 (0.72)	3.47 (1.12)

#### Specific observations

*Core competencies: Regarding the competency of relation with patients*: All graduates were ranked low by supervisors and similarly *for relation with colleagues:* All were ranked low by supervisors except working effectively with other health professionals.

Furthermore, for *teaching, good practice, working within the system in the context of health care as well as professionalism,* all sub-competencies were ranked low by graduates and supervisors.

*Professional knowledge:* was favorably ranked in all aspects except that supervisors ranked the students low in knowledge of causes and progression of diseases, employing knowledge of non-communicable disease, common surgical conditions and common obstetrical and gynaecological conditions. Furthermore, *Practical/Clinical Skills:* All were also ranked low by supervisors except for universal precaution.

**Fig. 3:**
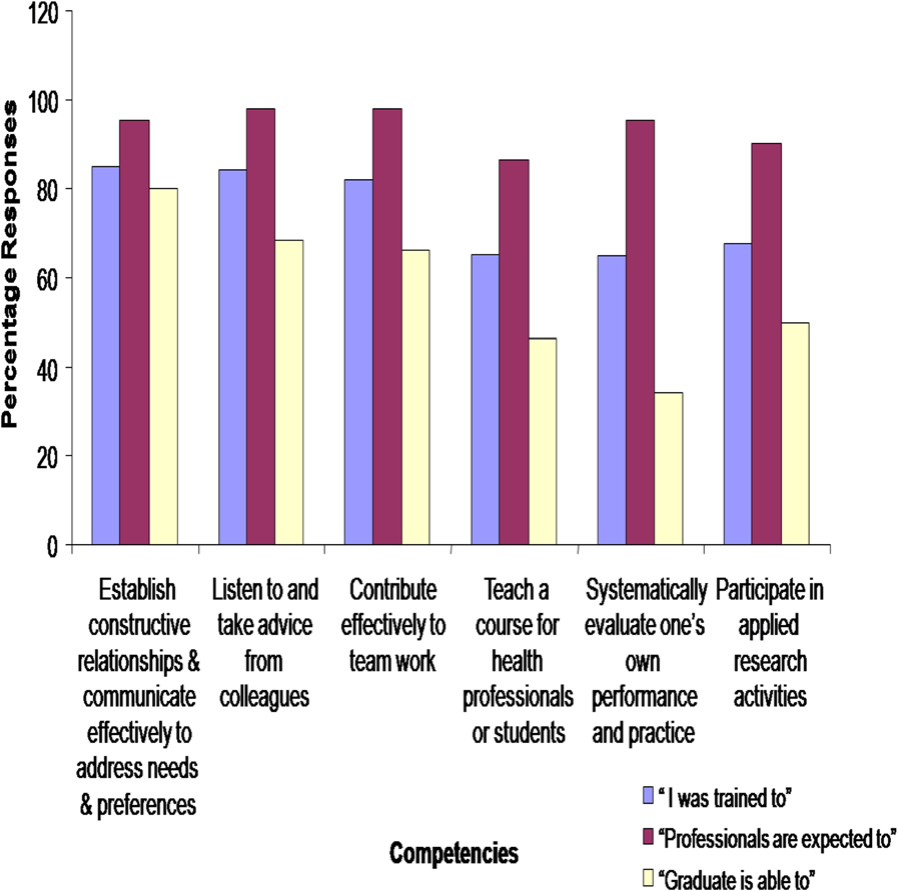
Bar Chart showing the assessment of some of MUHAS core competencies by graduates themselves in relation to their training; as well as what others expect from them including what they are finally able to perform. Generally, expectations seemed to be higher than the training they received as well as the competencies and skills they were able to demonstrate in practice

As regards the assessment of some of MUHAS core competencies by graduates themselves in relation to their training; as well as what others expect from them and what they are finally able to perform, it was generally observed that expectations seemed to be higher than the training they received as well as the competencies and skills they were able to demonstrate in their practice (Fig. 3).

*Graduates opinions of courses undertaken at MUHAS:* In the opinion of graduates, nine courses namely: Anatomy, Biochemistry, Behavioral Sciences, Development Studies, Introduction to Clinical Medicine, Nutrition Field Project, Clinical Pharmacology, Introduction to Clinical Medicine, Orthopaedics & Traumatology did not prepare them well enough for their professional needs after graduation.

In the opinion of graduates, the courses that they studied in earlier semesters at MUHAS did not prepare them well to undertake the following courses: Behavioral Sciences, Development studies, Microbiology and Immunology, Introduction to Clinical Medicine, Pathology, Epidemiology and Research methodology, Nutrition Field Project, Forensic Pathology, Clinical Pharmacology, Medical Ethics, Orthopaedics & Traumatology.

In the opinion of graduates, Seminars/Tutorials, Laboratory Skills/Practicals, Theatre skills, Outpatients clinics, Family Case Studies, visits/excursions and Self Reflection were rated as less useful teaching methods compared to Lectures, Teaching Ward Rounds, Elective Studies, Field Work and Presentations.

ICT and Library facilities were not considered to meet the students learning needs as well as Clinical logbooks were ranked low as a method of assessment. Furthermore, Teachers were ranked less favorably in all assessed roles including professional role modelling by teachers, accessibility of teachers outside scheduled teaching sessions, monitoring of students, teaching sessions, academic advice given and nature of students’ relationship with teaching staff. Of these, teachers were ranked lowest for accessibility of teachers outside scheduled teaching sessions (Table 5).

**Table 5:** Graduates opinions regarding the learning environment

*n* = 147				
Figures in cells are based on a Likert Scale from 1 to 5, where 1 = strongly disagree and 5 = strongly agree		Numbers	Percentage agree	Mean (SD)
“How useful were the following teaching method to helping you learn?”	Lectures	104	79.90 %	4.07 (0.87)
	Seminars/Tutorials	83	69.90 %	3.90 (1.02)
	Laboratory skills/Practicals	118	51.70 %	3.54 (1.11)
	Theatre skills	123	45.50 %	3.39 (1.33)
	Teaching ward rounds	96	80.60 %	4.12 (0.99)
	Outpatients clinics	88	66.20 %	3.80 (1.02)
	Family case studies	117	47.20 %	3.37 (1.19)
	Elective studies	118	75.00 %	4.04 (0.91)
	Field work	121	78.60 %	4.17 (0.92)
	Presentation	90	86.80 %	4.32 (0.82)
	Self reflection	99	68.30 %	3.89 (1.03)
	visits/excursions	104	31.70 %	3.39 (1.58)
“The following meet my learning needs”	Computer lab	119	21.50 %	2.27 (1.35)
	Libraries	105	60.10 %	3.46 (1.23)
	Internet Access	99	23.90 %	2.39 (1.37)
“How useful were the following assessment methods to your learning?”	Continuous assessments	59	82.20 %	4.17 (0.92)
	Final examinations	108	76.70 %	4.14 (0.91)
	Multiple choice	111	72.10 %	3.88 (0.93)
	Essays	110	76.00 %	3.96 (0.95)
	Short answer s	113	80.40 %	4.06 (0.87)
	Oral examination	117	68.00 %	3.81 (1.23)
	Field work/projects	109	72.00 %	4.16 (0.90)
	Clinical/practical examination	121	84.80 %	4.28 (0.88)
	Clinical logbooks	135	40.20 %	3.24 (1.37)
	Research report	107	70.50 %	3.88 (0.99)
	Presentations	121	81.80 %	4.22 (0.94)
“Rate MUHAS teachers in the following roles”	Professional role modelling by teachers	126	47.90 %	3.24 (1.13)
	Accessibility of teachers outside scheduled teaching sessions	114	19.00 %	2.34 (1.15)
	Monitoring of students	95	31.50 %	2.77 (1.20)
	Teaching sessions	128	57.90 %	3.51 (1.02)
	Academic advice given	45	31.70 %	2.83 (1.28)
	Nature of your relationship with teaching staff	34	23.30 %	2.68 (1.25)

### Qualitative studies results

Students chose a wide variety of courses to comment on. The distribution of the courses is represented in Tables 6 which shows courses that had the most respondents.

**Table 6:** Graduates opinions regarding courses undertaken at MUHAS

Figures in cells are the means of Likert Scale from 1 to 5, were 1 = strongly disagree and 5 = strongly agree		This course prepared me for my current professional needs		MUHAS prepared me to take this course	
		*n* = 147		*n* = 147	
	Number	Percentage agree		Percentage agree	
Anatomy	104	70.9 %	3.96 (1.11)		
Biochemistry	83	56.5 %	3.59 (1.18)		
Medical Ethics I	118	80.0 %	4.11 (1.05)		
Physiology	123	83.6 %	4.30 (0.90)	75.90 %	4.04 (1.13)
Behavioral Sciences	96	65.0 %	3.82 (1.04)	56.50 %	3.50 (1.33)
Development studies	88	60.0 %	3.69 (1.15)	50.00 %	3.38 (1.35)
Microbiology/Immunology	117	79.3 %	4.21 (0.96)	74.80 %	3.97 (1.06)
Parasitology/Medical Entomology	118	80.0 %	4.19 (0.94)	79.00 %	4.03 (1.06)
Clinical Physiology	121	82.0 %	4.24 (0.97)	78.80 %	4.16 (1.04)
Development studies	90	61.0 %	3.64 (1.13)	59.00 %	3.61 (1.28)
Introduction to Clinical Medicine	99	67.6 %	3.83 (1.24)	67.80 %	3.84 (1.32)
Pathology	104	70.8 %	4.02 (1.05)	73.30 %	3.99 (1.17)
Epidemiology & Research Methods	119	81.2 %	4.17 (0.92)	63.60 %	3.76 (0.11)
Nutrition Field Project	105	71.3 %	3.95 (1.14)	72.00 %	3.95 (1.12)
Introduction to Clinical Medicine	99	67.4 %	3.84 (1.27)	71.40 %	3.92 (1.24)
Forensic Pathology	59	39.9 %	2.91 (1.45)	45.40 %	3.13 (1.46)
Clinical Pharmacology	108	73.5 %	3.96 (1.12)	70.10 %	3.86 (1.22)
Management of Disease I	111	75.7 %	4.04 (1.12)	79.80 %	4.08 (1.09)
Medical Ethics II	110	75.0 %	4.06 (1.09)	70.00 %	3.88 (1.20)
Medical Ethics III	113	76.6 %	4.10 (1.04)	71.70 %	3.96 (1.14)
Management of Disease II	117	79.6 %	4.19 (1.00)	71.70 %	4.24 (1.00)
Community medicine	109	74.1 %	4.00 (1.12)	76.10 %	4.01 (1.11)
Paediatrics & Child Health	121	82.0 %	4.20 (1.08)	77.80 %	4.13 (1.10)
Obstetrics & Gynaecology	135	92.1 %	4.50 (0.78)	85.30 %	4.36 (0.94)
Elective Period	107	72.8 %	4.05 (1.01)	76.40 %	4.07 (1.08)
Surgery	121	82.0 %	4.21 (0.97)	80.20 %	4.15 (1.01)
Internal medicine	126	85.4 %	4.35 (0.98)	80.00 %	4.19 (1.07)
Surgical Specialties	114	77.5 %	4.12 (1.04)	76.30 %	4.14 (1.10)
Orthopaedics & Traumatology	95	64.5 %	3.78 (1.25)	66.40 %	3.82 (1.28)
Psychiatry	128	86.8 %	4.45 (0.81)	81.10 %	4.21 (1.00)

Themes with 3 % of more of responses are presented as individual themes, and other responses compiled into an “other” category. Responses were then analyzed into major thematic categories of: professors/staff, over-enrolment and class size, facilities and educational materials, curricular content, and duration of courses.

The quality of the responses varied by question; for course strengths, 12 % of answers were coded as generic and contained statements such as “the course was good”. The responses for weaknesses and improvements were much more content rich with only 2–4 % of answers coded as general. Answers were coded as generic when they did not contain any specific references and when they used undescriptive adjectives such as “good” or “bad”.

#### Professors/Staff

A large percentage of students’ comments referred to employees of MUHAS, particularly the lecturers, tutors and support staff. About 49 % of students mentioned characteristics of professors as strengths of a course, and 315 as weaknesses. Their comments focused on the availability of professors, as well as their attitudes and their ability to convey the material. They also focused on the role of the tutors and teaching assistants. Students spoke about the importance of engaged professors, their attendance in class and in the clinic, and consistent delivery of the material. Finally organization and commitment were also important qualities students looked for in their professors. Positive attitude, friendly demeanor and approachability were noted with high praise. Some of the quotes from students regarding Professors strengths included:

- “Lecturers were well organized and available”
- “Lectures are well narrated”
- “Lectures were delivered with effectiveness”
- “Lecturers were committed to teaching”
- “Lecturers were committed and subject interesting, focused the current problems e.g. avian flu epidemic”
- “Lecturers- highly committed and very friendly”
- “Nice lecturers, well informed, well prepared”
- “Tutors were available and very responsive” and
- “Lecturers tried their best to deliver information, lectures were participatory.”

Weaknesses focused on a lack of availability, attendance, particularly in the clinical rounds and finally on the attitude of the professors. Students noted that professors were often not present in class or in the practicals. Students perceived that faculty were overworked and overcommitted and linked this to the lack of faculty within a department. Several other students mentioned that perhaps, if the professors spent less of their time doing research or working in the private sector, they might have more time to teach. Some of the quotes from students regarding Professors weaknesses included:

- “Poor teaching methods and lack of participatory teaching”
- “No good cooperation between lecturer and employer”
- “No permanent teacher for this subject”
- “Few supervisors were monitoring and guiding us”
  “Some of the lecturers were showing favoritism towards some students”
- “Lecturers did not engage fully on delivering materials, so theoretically lecturers were not friendly with students”
- “One lecturer provides too much load for him”
- “Lecturers are so harsh and very embarrassing and lack good cooperation with their students”
- “Every lecturers needs his/her own way of examination to patients (not following the reference books)”
- “During ward round teachers are not around most of the time”

Furthermore, students found that many weaknesses in regards to the professors were linked with over-enrolment and a lack of formal training in education. Suggestions to improve the learning environment included formal classes in education methods and hiring of support staff such as tutors and teaching assistants. Likewise, suggestions for improved attendance of teachers included hiring more lecturers and creating a system that would hold teachers accountable for their attendance.

#### Over enrolment and class size

The number of students present in the learning environment was extremely important to MUHAS students. About 18 % of students cited over-enrolment specifically as a problem. Only 3 % of students mentioned small class size as a strength of a course they had taken at MUHAS.

Weaknesses and suggestions for improvements focused on decreasing class size especially in regards to lab work and practical, hands-on learning. Solutions to the problems of over enrolment were overwhelmingly to increase facilities, increase the number of classes to reduce size of class and to employ more professors and teaching staff. Other suggestions included enlarging facilities, especially labs; and improving teaching materials and resources to address the large number of students. Students also recommended creating study groups and lab groups of ~6 or fewer students. Very few students mentioned decreasing enrolment, but rather increasing the university’s capacity.

#### Facilities and educational materials

Students frequently mentioned educational materials, such as books, handouts, study materials, access to models, reference materials and books and technology in their responses. While no students mentioned the availability of educational supplies as acourse strength, 18 % cited it as a weakness, and 17 % made recommendations based on improving access to supplies. In addition to materials, facilities including labs, audiovisual rooms, and cadaver rooms were brought up in all three sections. Classes were commended for having materials and facilities that were adequate for learning. There was a high frequency of responses in regard to practical and clinical work, especially in regards to the availability of lab materials and access to facilities. Many suggestions for improving facilities suggested expansions to adjust for over enrolment. In addition to facilities, improved, up-to-date learning material was deemed critical to improving the classes.

#### Duration of courses

The duration of courses was especially important in regards to the weaknesses and suggestions for improvement. Many of the students suggested increasing the amount of time in their schedules for specific, information-rich courses, as well as rearranging the syllabus to include more time for practical and clinic-based learning. Other suggestions included extending classes to include a second semester. Appropriate time for classes was deemed necessary by the students in all three categories.

#### Other suggestions

- “(have someone else complete) arrangements to be done (for) seeking patients instead of students seeking patients”
- “Lower registration fee for volunteer patients”
- “Establish a cardiology unit at MUHAS with basic diagnostic and interventional facility”
- “Reduce the number of appointments to patients and that will encourage more patients”
- “Focus to be made on microbiological problems in our region”
- “The MUHAS (should) have its own ultrasound machines instead of depending on those of MNH”
- “Use computers and PowerPoint methods, abandon old methods of teaching using transparent”
- “Lecturer – student communication should be improved”
- “Lecturers should adhere to timetable set”
- “Machines and materials are required to appreciate what are taught in theory”
- “Improve standard in wards for patients and health professionals”

#### Specific course comments

Graduates gave the following opinions regarding various courses they attended at MUHAS:

**For basic sciences**, strengths included Learning and reading materials were available, thorough professors that were also enthusiastic, cooperative, and friendly as well as good course organization. Weaknesses included overcrowding, lack of enough lab supplies lecturers were not often present in lab section and lack of enough time. Recommendations included more instructions for lab work, increase the time, increase the number of tutors, increase access to teaching materials, improve lab equipment, Make slides and other materials available for study and expand some of the courses to two semesters.

**For clinical sciences**, strengths included engaged, available, interesting and committed lecturers, ward rounds were very useful and taught very well, large number of staff and well prepared teaching materials and also professors were well organized and gave good demonstrations. Weaknesses included overcrowding in the ward, Not enough orientation to clinical and surgical skills (for example limited exposure to skills such as stitching or normal delivery), inappropriate and subjective exams, biased assessments, poor time schedule for exams and lack of enough time to cover all the materials. Recommendations included improving the learning environment and the attitudes of the professors, increasing the amount of time spent on bedside teaching and increase the supervision in clinic, add a two semester teaching program, change mode of teaching and examination, require senior teachers to attend ward rounds, increase the number of lectures, have the professors follow standard book protocols, group students to facilitate learning, encourage students to participate in procedures as well as increase theatre time and rotation time.

### Focused group discussions results

#### Summary

A series of focus groups were conducted in the summer of 2009 with recent graduates from the Muhimbili University of Health and Allied Sciences (MUHAS).

The discussions explored graduates perceptions of the curriculum and possible improvements. Discussion focused on over enrolment, facilities, supplies and faculty, preparation for practical work and fieldwork experiences. Students provided very specific recommendations for each health science school, which is included in a section of this report.

Discussions were semi-structured and included prompt questions soliciting opinions on the challenges of the curriculum and how students propose to improve them Focus group discussions were recorded and transcribed. Data was then coded and analyzed according to major themes.

In order to provide information for the curriculum revision, this report focuses mostly on constructive criticism directed at areas in need of improvement whereas in the focus group discussions focused both on praise and criticism. Major themes of discussion included facilities and supplies, over enrolment, educational time frame, theoretical preparation for practical work, experiential learning and fieldwork, communication, course load, skills and faculty. These themes are discussed below, followed by specific improvements that were proposed in each school.

### Thematic analysis results

#### Facilities and supplies

All groups, aside from the environmental health science students, mentioned facilities and supplies during their discussions. Facilities refer to spaces for learning such as classrooms, laboratories and clinical space for internships and equipment refers to educational materials, such as books and computers. Comments regarding facilities ranged from lack of seats and desks in classrooms, to requests for more clinical space. Over all five groups there was an equal frequency of comments about facilities and supplies. In particular, Medicine students mentioned a lack of technology and equipment. Most of the students concerns were directed at clinical facilities. Students reported a lack of clinical facilities that met the standards for their internships. The hospitals and clinics did not have the required departments that were required for their rotations. Within their fieldwork and rounds, students reported a lack of supplies and patients to work on. Finally, there were also comments on the lack of educational supplies such as media, copies of handouts and books. Some of the quotes from students regarding facilities and materials included:

##### Strengths

- “Good arrangement of firms where all students get same materials”
- “Lecturers were available, laboratory was well-equipped”
- “Dedicated lecturers. Availability of reading materials & Lecturers”
- “Illustration unit helped us in cadaver room”

##### Weaknesses

- “Had inadequate and old teaching material”
- “There are no models for teaching and based on skeletal anatomy mainly”
- “The materials especially books were not available we use past papers”

“The teaching system is very old and poor, no PowerPoint even the materials were not updated”

#### Over enrolment

Over enrolment was specifically mentioned by medical students. The increased enrolment was discussed most often in reference to lack of facilities and lack of equipment. Students from the Medical school spoke about roles of government and politicians in enrolment quotas.

“*To me may be the disappointments are seeing…the government is having the good idea of increasing the number of enrolling the number of MD students”* (Medical Student, p16).

A medical student mentioned the conflict between the need to increase medical workforce by increasing the number of medical students in the country and the lack of facilities and professors. One student mentioned the university’s inability to hire more professors to meet the need for the increased number of students. The students believe that the university has increased enrolment without increasing facilities, supplies and professors, which has decreased the quality of education.

*“(The) other thing is that I think the number of teachers, the number has been there since… the admission of those the years, the past years, may be they were taking fifty students but now they are taking about more than two hundred but the, still the number is limited*” (Medical Student, p20).

#### Educational time frame

Comments included lack of continuity or connection between subjects, repetition of material and a heavy course load. The observations regarding time were coupled with desire to gain the best education possible and were most often related to inefficient use of time or lack of time.

Students were not in agreement on the continuity and the flow of courses. Most of the groups had similar observations that there were no connections between the different courses. These comments were followed with some disagreement and generation of specific examples of subjects that did provide continuity.

The concept of efficiency was brought up often in reference to repetition within the curriculum. Repetition in the curriculum was particularly important to medical students. However, time and efficiency of learning were often linked in the students’ discussion. Some of the quotes from students regarding facilities and materials included:

- “Short period of rotation”
- “Very long course; is covered within a very short time”
- “Things were too condensed and became too exhausting”
- “Course duration was not consistent with course materials”
- “The programme has a lot of free time which can be used in other rotations”
- “Time/days selected for field work are too short compared to the work suggested to be done during the field project”

#### Theoretical preparation for practical work

One of the most important topics discussed frequency in every focus group was theoretical preparation for practical work. The biggest comment that was repeated in every school was that students did not feel that they were being adequately prepared for their clinical experience. Much discussion was directed at reordering the classes so that appropriate theoretical knowledge could be gained before clinical exposure. Students reported either being taught theoretical concepts after fieldwork, not having enough of theoretical training or not receiving any theoretical training. Most students shared the view that they were undertrained in the practical part of their education.

Though this viewpoint was a major concern, it was also shared with its inverse, receiving too much theory without the clinical background. Some of the quotes from students regarding the balance between theory and field/clinical/laboratory practice included:

- “Time/days selected for field work are too short compared to the work suggested to be done during the field project”
- “A lot of information and short time allocated”
- “Limited laboratory time and specimens”
- “Need enough time to practice”
- “Set enough time to practice all necessary procedures”
- “Machines and materials are required to appreciate what are taught in theory”
- “Short period of rotation”
- “More time should be allocated to these ward rounds”

This was echoed in all of the focus groups and demonstrates the delicate balance between theoretical and practical learning.

## Discussion

Recent medical graduates from the SoM who had undergone training through the semesterized programme, where thus assessed both quantitatively and qualitatively in order to gauge how much they felt they possessed competencies and skills including MUHAS core competencies (Additional file 3) as well as those specific for the MD degree (Additional file 3); and how much they felt enabled/empowered or disadvantaged by the curriculum they went through [2]. Furthermore, supervisors, co-workers, patients and employers were asked to evaluate how much graduates displayed those competencies and skills and whether they performed better or worse compared to graduates from other medical schools [2].

The overall goal was to develop competency-based curricula that are relevant to the national socio-economic development; respond to market demands and stakeholders needs; and conform to MUHAS vision and educational standards.

As regards the quantitative analysis it was revealed that with the exception of FOUR aspects, most competencies ranked low including teaching, health care system and good practice which ranked the lowest as can be seen in Table 4 and Additional file 4 and that there was reasonable concordance were rating was low. This generally implies that expectations seemed to be higher than the training the graduates received including the competencies and skills that they were able to demonstrate in their practice. However, the competency of Professional Knowledge was favorably ranked in all aspects except that, supervisors ranked the graduates low in knowledge of causes and progression of diseases, employing knowledge of non-communicable disease, common surgical conditions and common Obs & Gyn conditions. Nevertheless, this low ranking may be representative of a graduate population who had undergone mostly the knowledge-based curriculum in contrast to the new competency-based learning which was being introduced during this index review. Furthermore, in-depth questionnaire analysis generally revealed some strengths including teachers’ enthusiasm, organization as well as good demonstrations. Some of the weakness included inavailability and lack of enough faculty as well as poor teacher-student relationship. Focused group discussions also revealed lack of equipment and facilities, over-enrolment and faculty deficiencies as seen above. The findings of the index tracer study directly informed and guided the ongoing curriculum review exercise including introduction of MUHAS core as well as MD-specific competencies and skills, repackaging, full modularization, increased inter-professional interactions and teamwork, other improvements [2]. A good example is the reorganization/restructuring of the MD programme from 3 years basics and 2 years clinical sciences formerly (Additional file 5) which is now reversed to 2 years basics and 3 years to accommodate the one of the most important findings of the tracer study-need for improved clinical skills and competencies and hence more time for clinical sciences (Additional file 5). This goes alongside the re-introduction of junior and senior clinical rotations to allow for repeated learning which is pivotal to clinical apprenticeship. Other important outcomes of this tracer study are the extension of some basic science courses including anatomy, biochemistry, physiology and pathology to two semesters (Additional file 5). The MD curriculum has been a subject of multiple minor cosmetic changes ever since. These are based on implementation challenges and are not the scope of this study. This semesterized, modularized and competency-based curriculum has been in implementation will now be almost due for a subsequent revision and thus it is important to publish these findings in order also to inform the next Tracer Study and Curriculum Review.

## Conclusions

Thus the second tracer study as well as curriculum review exercise were conducted and whose results allowed for the repackaging of the MD curriculum, the introduction of full modularization as well as that of competency-based learning within SoM and MUHAS generally. It is envisioned that these tracer study findings will improve teaching and learning at MUHAS and lead to increased output of appropriately trained health professionals to fill the big gap in human resources for health (HRH) in the country. This will also promote interprofessional interactions and teamwork geared towards patient care as well as health promotion. The revised curriculum was also submitted for processing through the TCU for accreditation as required by the National Regulatory Authority. It is recommended that programmes, courses, modules as well as topics should emphasize on the teaching and assessment of competencies and skills and that focus should shift from the emphasis on teaching (teacher-centered education) to learning (student-centered education). The fact that the new curriculum will soon be subject to another review necessitates the publication of these results so that new changes are informed by the current findings.

## Additional files

Additional file 1: SoM Postgraduate Programmes During the Tracer Study. (DOC 29 kb) Additional file 2: Distribution of Respondents by School. (DOC 264 kb) Additional file 3: List of General and Specific Program Competencies. (DOC 36 kb) Additional file 4: Quantitative Tables. (DOC 234 kb) Additional file 5: MD Programme Summaries. (DOC 109 kb)

## Abbreviations

ALP: Academic Learning Project
DCEPD: Directorate of Continuing Education and Professional Development
ICT: Information, communication and technology
MD: Doctor of Medicine
MMed: Master of medicine
MUCHS: Muhimbili University College of Health Sciences
MUHAS: Muhimbili University of Allied and Health Sciences
SoM: School of medicine
TCU: Tanzania Commission for Universities
UCSF: University of California San Francisco
