# Supplementary material for: The Doctor of Medicine curriculum review at the School of Medicine, Muhimbili University of Health and Allied Sciences, Dar es Salaam, Tanzania: a tracer study report from 2009
Source: BMC Med Educ. 2016 Aug 25;16(1):223. doi: 10.1186/s12909-016-0745-7 (PMC5000497; doi:10.1186/s12909-016-0745-7)
Supplement: Additional file 1: — SoM Postgraduate Programmes During the Tracer Study. (DOC 29 kb) [file 12909_2016_745_MOESM1_ESM.doc]

## Additional file 1***:***

## ***List of SoM Postgraduate Programmes During the Tracer Study***

## ***Post-graduate degrees:***

## ***Master of Medicine (MMed) in*:** 1. Anesthesiology (MMed Anaesthesiology) 2. Anatomical Pathology (MMed Anat Path) 3. Clinical Oncology (MMed Clin Oncol)

4. Emergency Medicine (MMed Emergency Medicine)

## 5. Haematology and Blood Transfusion (MMed Haematology/BT) 6. Internal Medicine (MMed Int Med) 7. Microbiology and Immunology (MMed Micro/Imm) 8. Obstetrics and Gynecology (MMed Obs/Gynae) 9. Ophthalmology (MMed Ophthal) 10.Orthopedics and Traumatology (MMed Ortho/Trauma) 11. Otorhinolaryngology (MMed Otorhinolaryngology) 12. Pediatrics and Child Health (MMed Paed/Child Health) 13. Psychiatry (MMed Psych) 14. Radiology (MMed Radiology) 15. Surgery (MMed Surgery)

## 16. Urology (MMed Urology) **Master of Science (MSc) in:** 1. Anatomy (MSc Anatomy) 2. Clinical Pharmacology (MSc Clin Pharm)

## 3. Physiology (MSc Physiology) 4. Biochemistry

5. Clinical Psychology

***Master of Science (MSc) Superspecialization in:***

## *****1*****. Cardiology (MSc Cardiology) 2. Nephrology (MSc Nephrology) 3. Orthopedics and Traumatology (MSc Ortho/Trauma) 4. Neurology (MSc Neurology)

5. Neurosurgery (MSc Neurosurgery)
6. Haematology and Blood Transfusion (MSc Haematology)

7. Respiratory Medicine

***Doctor of Philosophy (PhD)***
