# Supplementary material for: The Doctor of Medicine curriculum review at the School of Medicine, Muhimbili University of Health and Allied Sciences, Dar es Salaam, Tanzania: a tracer study report from 2009
Source: BMC Med Educ. 2016 Aug 25;16(1):223. doi: 10.1186/s12909-016-0745-7 (PMC5000497; doi:10.1186/s12909-016-0745-7)
Supplement: Additional file 2: — Distribution of Respondents by School. (DOC 264 kb) [file 12909_2016_745_MOESM2_ESM.doc]

**Additional** **file 2: QUESTIONNAIRES**

**MUHIMBILI UNIVERSITY OF HEALTH AND ALLIED SCIENCES**

# GUIDELINES FOR TRACER STUDIES FOR MUHAS GRADUATES OF 2004-2008

**SURVEY FOR EMPLOYERS OF GRADUATES FROM MUHIMBILI UNIVERSITY OF HEALTH AND ALLIED SCIENCES**

Dear Employer

The Muhimbili University of Health and Allied Sciences (MUHAS) recognizes the potential contribution of end-users of its graduates have on improving its academic programmes. In order to tap this, the University is conducting a tracer study whose results will guide in reviewing its academic programmes.

The University is requesting you to participate in this study by filling in a questionnaire and respond to the few questions. The main areas include a broad overview concerning the knowledge and skills, performance, sense of cooperation with other workers, responsibility, professional career development and attitude towards work of the MUHAS graduates working in your organization.

MUHAS assures you that your responses will only be used for the purpose of this survey. In the description of results of this survey no identification of individual persons will be made.

your information will be TREATED WITH strict confidentiality.

MUHAS will be pleased to send you an overview of the most important results if you wish.

Thank your very much for your kind participation.

Please provide your E-MAIL or POSTAL address for receiving study results at the end of the questionnaire.

**Participant ID number.** └─┴─┘└─┴─┘

**(Please do not write any name)**

**GENERAL QUESTIONNAIRE FOR EMPLOYERS OF GRADUATES FROM MUHAS**

**Serial number:……………………………………**

##### Notes on the Completion of the Questionnaire

You are requested to answer each of the following questions and tick the most appropriate answer where needed. In some cases you are asked for numbers only (└─┴─┘), while in others a space is left for you to write an answer ( .................... ).

Should there not be enough space for your answers, please include an additional sheet of paper.

##### CHARACTERISTICS OF THE EMPLOYING ORGANIZATION

###### What type is your Organization?

- Government/public agency
- Non-governmental/private agency

###### Please specify the field in which the organization can be best classified. *Please tick once.*

01 National/Referral Hospital

02 Regional hospital

03 District hospital

04  Health centre

05  Private/faith based hospital

06  MDA’s (ministries/Departs/Agencies)

07  Non-Government Organization

Other: …………………………………………

*(Please specify)*

###### 3 How many people are employed in your organization?

└┴┴┴┴┘Employees

###### How many MUHAS MD graduates are employed in the organization?

└┴┴┴┴┘ Total

└┴┴┴┴┘Female

└┴┴┴┴┘Male

###### How many MUHAS Specialists are employed in the organization?

└┴┴┴┴┘ Total

└┴┴┴┴┘Female

└┴┴┴┴┘Male

##### B. RECRUITMENT PROCEDURES AND CRITERIA

Please indicate the way in which you select your (e.g. Nurses, Doctors, Teachers, Pharmacists, Nurse Tutor Environment Health specialists officers, etc) in recruitment procedures and what experience you have gained.

###### How important in general are the following aspects for the recruitment of graduates?

###### *Please indicate the importance of each of the following aspects with the help of the 5-grade scale.*

very

import- Least

tant important

1 2 3 4 5

     Computer proficiency

     English fluency

<1>      Field of study<1>

<1>      Grades of examinations at the university

<1>      Ability to express oneself in writing<1>

<1>

<1>      Reputation of candidate’s university<1>

<1>

<1>      Recommendations/references

<1>      Results of Interviews

<1>      Candidate’s world view

<1>      Other:………………………



<1> *(Please Specify)*

##### C. ORIENTATION PERIOD

In general, subsequent to university studies a period of orientation is inevitable in order to acquire all the necessary skills for the specific tasks required in the new job. The following questions aim at the duration and concept of the orientation period in the organization.

###### 6. In your opinion, how long does it take before newly employed MUHAS graduates who have just finished their university studies are able to carry out professional tasks properly?

01- 03 months

04 – 06 months

07 – 09 months

10 – 12 months

More than 12 month …………………………

<3>

**7.**  **For orientation times required by different groups of MUHAS graduates vary, in your opinion what are the decisive reasons for this?**

…………………………………………………….

…………………………………………………………………

…………………………………………………………………

###### 8. From your experience, up on recruitment MUHAS graduate usually, (*Please tick one item only)*

1  Start with normal tasks without any support

2  Seek advice/help from experienced colleagues

3  Seek advice/help from superiors

4  Participate in an extensive formal trainee programme/training course

5  Other: ……………………………….

*(Please specify****)***

###### 9. What kind of knowledge and abilities are usually acquired during the orientation period of a MUHAS graduate? (*Multiple responses possible)*

 Knowledge of the organizational structure of the institution or of single sections

 Application-of relevant management Information Systems (MIS)

 Knowledge in the management

- Getting acquainted with the facilities, equipments and environment
- Familiarization will colleagues and responsibilities

 Other: …………………………………………

*(Please specify)*

###### 10. Are MUHAS graduates well prepared to undertake future management posts when they start to work in the organization?

- Yes
- No

##### d. FURTHER PROFESSIONAL DEVELOPMENT

*The questions in this section deal with the contents, structures, and basic conditions of further professional training offered in the organization*

###### 11. Does the organization offer any opportunities for further professional development?

- Yes
- No

If yes, where does the training take place?

***(MULTPLE RESPONSE POSSIBLE)***

- Locally within the organization
- locally outside the organization
- Abroad

If Local, list the courses offered.

…………………………………………….

……………………………………………..

……………………………………………..

###### 12. To what extent does the organization support the participation of MUHAS graduates in further professional orientation by releasing them from the organization specific tasks?

to a

very high to a very

degree low degree

1 2 3 4 5

<1>

###### 13. If further professional development for MUHAS graduate is offered by external (education) institutions, to what extent are costs paid by the organization (attendance fees, accommodation, travelling costs)?

to a

very high to a very

degree low degree

1 2 3 4 5

<1>     

###### 14. Does the organization offer grants for courses of further study ending with an academic award (secondary studies, research studies, masters or PhD)?

- Yes
- No

    

##### e. COMPETENCES REQUIREMENTS OF GRADUATES FROM MUHAS

###### 15. What are competences requirements of graduate from MUHAS? (*Please tick one item of “major task” and, if necessary, further items of “additional activities”.)*

Major Additional

Task activities

01   Curative services

02   Diagnostic services

03   Counselling services

04   Rehabilitative services

05   Interaction with other Professionals

<1>06   Settlement/preparation of accounts

<1>07   Supervision

<1>08   Training

<1>09   Export

<1>10   Advisory services/consulting

<1>11   Maintenance and repair of facilities

<1>12   Controlling

<1>13   Data processing

<1>14   Development/experiments

<1>15   Finances

<1>16   Research and development

<1>17   Estimating/costing

<1>18   Management

<1>19   Marketing

<1>20   Market research

<1>21   Materials management

<1>22   Measurement and testing technology<1>

<1>23   Standardization and licensing

<1>24   Emotional Intelligence

<1>25   Planning of functions/use of buildings/plants

<1>26   Planning and organizations

<1>27   Project management<1>

<1> <1>

<1>

<1>28   Processing development

<1>29   Sales Management

<1>30  

Other::……………………………………………..

###### 16. Do MUHAS graduates in the organization take on tasks which could be performed by technicians or skilled workers (supporting staff)

<  Yes

1  No PLEASE GO TO QUESTION 21

###### 17. If MUHAS graduates in the organization have to take on professionally untypical tasks, what are the decisive reasons for this? *(Multiple responses possible*.)

 There is a lack of technicians/skilled workers

- MUHAS graduates are working in jobs which are usually occupied by people without a University degree.
- MUHAS graduates are given these tasks during their training period only in order to enlarge their practical experience.

 MUHAS graduates are given these tasks outside their training period also in order to train other persons in these fields

 Other:………………………………….

###### 18. How do you assess the knowledge and ability of MUHAS graduates in the following fields?

to a

very high to a very

extent low extent

1 2 3 4 5

<1>      General performance

<1>      Teaching skills

<1>      Counselling skills

<1>      Handling emergencies

     Disaster Management

<1>      Research competence

<1>      Cooperation with others workers

<1>      Punctuality

<1>      Willingness to do extra work hours

<1>      Customers’ satisfaction of services rendered by most of them

<1>      Interaction with other workers

<1>      Interaction with patients

<1>      Administrative skills

<1>      Professional confidentiality

<1>      Knowledge of the English language

###### 19. How do you assess the non-professional oriented attributes of MUHAS graduates in the following fields?

to a

very high to a very

extent low extent

1 2 3 4 5

<1>      Willingness to learn

<1>      Ability to solve problems

<1>      Reflective capability

<1>      Ability to express yourself in writing

<1>      Unconventional thinking

<1>      Willingness to perform/commitment

<1>      Ability to coordinate

<1>      Ability to improvise

<1>      Reliability

<1>      Ability to work under pressure

<1>      Independence

<1>      Sense of responsibility

<1>      Creativity

<1>      Economic reasoning

<1>      Determination

<1>      Loyalty to the institution and its objectives

<1>      Persistence

<1>      Ability to cooperate

<1>      Assertiveness

<1>      Self-confidence

<1>      Adaptability

<1>      Empathy

<1>      Leadership qualities

<1>      Initiative

##### f. PROSPECTS

These questions deal with changes in the organization

###### To what extent the following attributes MUHAS graduates contributes to the co-function of your institution?

| To a very high extent | | To a very low extent | | |  |
| --- | --- | --- | --- | --- | --- |
|  |  |  |  |  | Practical skills (experience) |
|  |  |  |  |  | Clinical skills |
|  |  |  |  |  | Diagnostic skills |
|  |  |  |  |  | Advisory skills |
|  |  |  |  |  | Reaction to emergency calls |
|  |  |  |  |  | Punctuality |
|  |  |  |  |  | Human relations |
|  |  |  |  |  | Provider of health care |
|  |  |  |  |  | Ethically and legally bounded |
|  |  |  |  |  | Professional Role Model |
|  |  |  |  |  | Communicator of health issues |
|  |  |  |  |  | Abilities planning and evaluation |
|  |  |  |  |  | Politically aware of the changes influencing health of the communities |
|  |  |  |  |  | Advocating of issues related to health at place of work and community |
|  |  |  |  |  | Advisor of health cultures |
|  |  |  |  |  | Knowledgeable coordinator of community resources |
|  |  |  |  |  | Facilitates learner development |
|  |  |  |  |  | Relationship with colleagues |
|  |  |  |  |  | Abilities in counselling |

21. Rate to what extent the following abilities possessed by graduates from MUHAS satisfies the needs of institution?

| To a very high extent | | To a very low extent | | |  |
| --- | --- | --- | --- | --- | --- |
|  |  |  |  |  | Applying approaches to effective leadership and management |
|  |  |  |  |  | Managing resources of human, money, material, time and space |
|  |  |  |  |  | Managing change and conflicts at work place |
|  |  |  |  |  | Participating in development of the organization |
|  |  |  |  |  | Planning and implementing programmes and projects of the organization |
|  |  |  |  |  | Participating in developing policies, standards and protocols of the organization |
|  |  |  |  |  | Participating in evaluating programmes and projects under the organization |
|  |  |  |  |  | Organizing environment to suit objectives of the organisation |
|  |  |  |  |  | Coordinating organizational functions |
|  |  |  |  |  | Conducting research |
|  |  |  |  |  | Other (*Specify*) |
|  |  |  |  |  |  |

###### 22. In your opinion, will your institution continue to employ graduates from MUHAS?

<  Yes

1  No

***(Please give reasons for your response)***

##### g. PERSONAL BACKGROUND OF THE INTERVIEWEE

Finally, we would like some brief information about your personal background

###### 23. Your position in the establishment

…………………………………………………

……………………………………………………………

……………………………………………………………

***Thank you very much for your kind co-operation.***

*Please enter your name and address or name and address of your employer in the appropriate space in order to enable us to send you - if you wish - an overview of the results of this survey.*

✂ This slip will be cut off (for data protection) immediately after receipt of the questionnaire ✂

 please tick this box if you are interested in receiving the report of the results

|  | **Own address** | **Address of the employer** |
| --- | --- | --- |
| **Name, first name (maiden name)** |  |  |
| **PO Box number** |  |  |
| **Email** |  |  |
| **Town** |  |  |
| **Country** |  |  |

# GUIDELINES FOR TRACER STUDIES

SURVEY OF GRADUATES OF

THE MUHIMBILI UNIVERSITY OF HEALTH AND ALLIED SCIENCES (MUHAS)

Dear Graduate

**The Muhimbili University of Health and Allied Sciences recognizes the potential contribution its graduates have on improving its academic programmes. In order to tap this,** MUHAS **is conducting a tracer study of its recent graduates, the results of which will guide the university in reviewing its academic programmes and undertaking a planned competency-based curriculum redesign..**

The University is requesting you to participate in this study by completing a questionnaire and responding to the questions. MUHAS is most interested in your views concerning the study programme while at the MUHAS, information regarding your employment situation, occupation and professional career plans.

MUHAS assures you that your responses will only be used for the purpose of this survey. No identification of individual persons will be used in the analysis and description of results of this study.

your information will be TREATED WITH strict confidentialITy.

MUHAS will be pleased to send you an overview of the most important results; please indicate your contact information below, if you wish to receive these results.

Thank you very much for your kind participation.

List your EMAIL or POSTAL address for receiving study results

_________________________________

**Participant ID number.** └─┴─┘└─┴─┘

**(Please do not write your name)**

**MUHIMBILI UNIVERSITY OF HEALTH AND ALLIED SCIENCES**

GENERAL GRADUATES’ QUESTIONNAIRE

##### INSTRUCTIONS on the Completion of Questionnaire

Please answer each of the following questions.

If answers are itemised, please tick the most appropriate answer. In some cases you are asked for numbers only (└─┴─┘) while in others a space is left for you to write an answer ( .................... ).

Should there not be enough space for your answers, please include an additional sheet of paper.

##### A. Course of StudY at THE SCHOOL OF MEDICINE

###### 1 Please, provide information on your study and training

|  | **Year** | **Area of  specialisation** |
| --- | --- | --- |
| M.D. degree  Start  Completion | <2> └─┴─┘  └─┴─┘ | N/A |
| Internship (if applicable  Start  Completion  Place | <2> └─┴─┘  └─┴─┘  ……………………………………………….. | N/A |
| 1^st^ Masters’ degree (if applicable)  Start  Completion  Place | <2>  <2> └─┴─┘  └─┴─┘  …………………………………………… | <2>  ......................... |
| 2^nd^ Masters’ degree etc. (if applicable)  Start  Completion  Place | <2>  <2> └─┴─┘  └─┴─┘  …………………………………………… | <2>  ......................... |

##### B. Socio-Biographic data

Dear Participant,


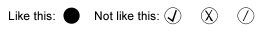
Thank you for taking part in the survey. It will take you about 30 minutes to complete. Please do not write your name on the questionnaire. Please fill in the bubbles completely, as shown in the following example:

***Section1: Study Participant ID and Demographics/Educational Background***

1. Age └─┴─┘ nearest year

1 (a). Gender: O male O female

|  |  |  |  |  |  |  |
| --- | --- | --- | --- | --- | --- | --- |

| 1. Profession: | O Medicine | O Dentistry | O Pharmacy | O Nursing | O Public Health/  Social Science | O Others (specify) |
| --- | --- | --- | --- | --- | --- | --- |

| 1. academic Level: | O Certificate | O Diploma | O Advanced Diploma | O 1^st^ Degree | O Masters | O Doctorate |
| --- | --- | --- | --- | --- | --- | --- |

| 1. Are you employed? | O Yes | O No |
| --- | --- | --- |

| 1. If employed, type of organization | O Public | O NGO | O Private | O Self Employed |
| --- | --- | --- | --- | --- |

| 1. Years working at the present Organization└─┴─┘ |
| --- |

| 1. Title and Professional Level | O Technical Staff | O Officer | O Manager | O Director |
| --- | --- | --- | --- | --- |

| 1. Citizenship | O Tanzanian | O Other (please specify): ______________ |
| --- | --- | --- |

1. Where is your workstation?

| O Dar Es Salaam | O other urban, including regional headquarters | O rural |
| --- | --- | --- |

##### C. Retrospective Assessment of Study at the SCHOOL OF MEDICINE

###### 1 How do you rate the study environment you experienced? Please rate each of the applicable options on a 5-point scale.

Ineffective very effective

1 2 3 4 5

<1>      Teaching quality of lecturers

    Structure of the programme

     Quality of the buildings

     Testing/grading system of examinations

     Chances to participate in research projects <1>

<1>      Opportunity of out of class contacts with teaching staff

<1>      Academic advice offered

<1><1>    Provision of supervised practical work experience

     Quality of equipment of the teaching hospitals

<1><1>      Availability of technical equipment (e.g. PC, laboratory equipment, etc.)

<1> <1>     Availability of teaching materials

<1>      Availability of relevant books in the library

     Availability of relevant books in the bookshops

     Affordability of relevant books in the bookshops

<1><1>      Assistance/advice for your final examination

<1>Other …????­­­­­­­­­­­­­­­­­­­­­­

###### 2. How effective were the following assessment methods employed by the school? Please rate each of the options on a 5-point scale.

Ineffective very effective

1 2 3 4 5

<1>      Continuous assessment

     Peer assessment.

<1>      Field project grades

<1>      Written end of semester examinations

<1>      Clinical logbooks/clinical assessment forms

<1>      End of semester oral examinations

<1>      Practical/clinical examinations

<1>      Elective period research report

<1>

###### 3 From the perspective of your current experience, having recently completed the MD programme, please:

###### 1) Rate how effectively each of the following courses serves your current professional needs.

|  |  |
| --- | --- |
|  |  |
| <1> | Ineffective very effective  1 2 3 4 5  <1>  <1><1>  **PRECLINICAL COURSES**        Anatomy  <1>      Physiology  <1>      Medical sociology  <1>      Biochemistry  <1>      Microbiology/Immunology  <1>      Psychology  <1>      Parasitology/Entomology  <1>      Pathology  <1>      Epidemiology  <1>      Biostatistics  <1>      Pharmacology  C  C  <1> **CL CLINICAL COURSES**        Surgery  <1>     Medicine  <1>      Paediatrics  <1>      Obstetrics/Gynaecology  <1>      Community Health  <1>      Psychiatry  <1>      ENT  <1>      Orthopaedics/Trauma  <1>      Ophthalmology  <1>      Forensic Medicine/Medical ethics  <1>      Development studies  <1>      Radiology  <1>      Anaesthesiology |
| <1> |  |

###### 2) From the lists above, please select at least TWO preclinical and at least TWO clinical courses that stand out for you and for which you can identify:

###### a) Specific strengths

###### b) Specific deficiencies and

###### c) Propose modifications for the MUHAS’s future competency-based curriculum

|  | **Name of Course** | **Strengths** | **Deficiencies** | **Proposed modification** |
| --- | --- | --- | --- | --- |
|  | Example:  Microbiol | Lecturers were consistently engaging and available | The labs were ill-equipped and overcrowded and disorganized | Develop more materials to prepare for labs in advance, so students can make more effective use of the time in the lab; consider assigning small groups to circulate around stations. |
| <1> |  |
| <1> |  |
|  |  |  |  |  |

**PRECLINICAL COURSES**

| **Name of Course** | **Strengths** | **Deficiencies** | **Proposed modification** |
| --- | --- | --- | --- |
| 1. |  |  |  |
| 2. |  |  |  |
| 3 |  |  |  |

**CLINICAL COURSES**

| **Name of Course** | **Strengths** | **Deficiencies** | **Proposed modification** |
| --- | --- | --- | --- |
| 1. |  |  |  |
| 2. |  |  |  |
| 3. |  |  |  |

**4. Please rate each statement twice. First, indicate how well MUHAS prepares its graduates and second, how important this is in your practice of medicine. The scale is SD (strongly disagree) D (disagree) N (no opinion) A (agree) SA (strongly agree)**

|  | **MUHAS prepares its graduates well to:** | | | | | **It is important for MUHAS graduates**  **in practice to:** | | | | |  |
| --- | --- | --- | --- | --- | --- | --- | --- | --- | --- | --- | --- |
|  | **SD** | **D** | **N** | **A** | **SA** | **SD** | **D** | **N** | **A** | **SA** |  |
| Teach students and other health care professionals | O | O | O | O | O | O | O | O | O | O |  |
| Communicate effectively to colleagues,  patients and their families | O | O | O | O | O | O | O | O | O | O |  |
| Formulate an optimal clinical plan for a  patient with a chronic illness | O | O | O | O | O | O | O | O | O | O |  |
| Demonstrate ethical standards in my  professional practice | O | O | O | O | O | O | O | O | O | O |  |
| Understand health care system to improve  health care outcomes / Assist patients in dealing  with system complexities | O | O | O | O | O | O | O | O | O | O |  |
| Apply administrative and resource  management skills. | O | O | O | O | O | O | O | O | O | O |  |
| develop employment for self and other health  professionals | O | O | O | O | O | O | O | O | O | O |  |
| Participate in creative and research activities | O | O | O | O | O | O | O | O | O | O |  |
| Plan an approach to prevent and manage  common health challenges | O | O | O | O | O | O | O | O | O | O | O |
| Contribute to effective leadership and  management in a health team / Demonstrate  leadership skills | O | O | O | O | O | O | O | O | O |  | O |
| Regularly seek information necessary to  improve patient care practices | O | O | O | O | O | O | O | O | O |  |  |
| Work collaboratively as a member of a health care team | O | O | O | O | O | O | O | O | O | O |  |
| Provide care sensitive to individuals from diverse backgrounds | O | O | O | O | O | O | O | O | O | O |  |
| Educate patients about their health and treatment plans | O | O | O | O | O | O | O | O | O | O |  |
| Use effective strategies for self-directed learning | O | O | O | O | O | O | O | O | O | O |  |

| OVERALL (Global assessment) | **Strongly Disagree** | **Disagree** | **No Opinion** | **Agree** | **Strongly Agree** |
| --- | --- | --- | --- | --- | --- |
|  |  |  |  |  |  |
| **I am satisfied with the quality of my education from MUHAS** | O | O | O | O | O |

**Other Remarks**

1. **Insert page numbers**
2. **A separate questionnaire needs to be developed for Graduates at Masters level from MUHAS**

**MUHIMBILI UNIVERSITY OF HEALTH AND ALLIED SCIENCES (MUHAS)**

# GUIDELINES FOR TRACER STUDIES FOR MUHAS GRADUATES OF 2004-2008

## QUESTIONNAIRE

**FOR THE END-USERS OF MUHAS GRADUATES**

***(Co-Workers)***

Good morning/afternoon/evening. My name is ___________________________.

The Muhimbili University of Health and Allied Sciences (MUHAS)

in collaboration with the Ministry of Health and Social Welfare (MOHSW) of Tanzania, is doing a survey on the contribution of end users of its graduates on its academic programmes.

This survey will help the University understand and document the findings that will guide in its curriculumreview process.

You as a stakeholder are requested to participate in this study responding to questions.

The main areas include a broad overview concerning the knowledge and skills, performance, responsibility, professional career development and attitude towards work of the MUHAS graduates working in this institution.

We assure you that your responses will only be used for the purpose of this survey. In the description of the findings of this survey no identification of individual persons will be made.

**YOUR INFORMATION WILL BE TREATED WITH STRICT CONFIDENTIALITY**.

We will be pleased to send you an overview of the most important results if you wish.

Do you have any questions about this survey or about your participation? Would you like to participate?

Thank your very much for your kind participation.

**Questionnaire no: |__|__|**

**A. GENERAL INFORMATION**

Region ………………………………………..

District ………………………………………

Name of Institution ……………………………………………..

**B. SCREEN QUESTIONS**

**Biographical Data**

***Please fill in the bubbles, following this example:***

***
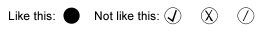
***

1. Gender: O male O female
2. Age: **|__|__|__|__|**

| 1. Profession: | O Medicine | O Dentistry | O Pharmacy | O Nursing | O Lab Technologist | O Public Health/  Social Science | O Others (specify) |
| --- | --- | --- | --- | --- | --- | --- | --- |

1. For how long have you been working in this institution?

**|__|__|** Months  **|__|__|** Years

O Yes O No

1. Do you have any recent MUHAS graduate (since 2007)?

O Yes O No

1. For how long have you worked with the MUHAS graduate?

: |__|__|__|__| Month : |__|__|__|__| Years

1. How do you assess the instructions provided by your colleague?

O Very clear O clear O ambiguous

1. What is the perception of patients/customers toward your colleague?

O Positive O Fair O Bad

1. To what extent are the following abilities and attitudes of your colleagues met

| Can’t assess | | Excellent | | Average | | Worse | |
| --- | --- | --- | --- | --- | --- | --- | --- |
|  |  |  |  |  |  |  | Practical skills (experience) |
|  |  |  |  |  |  |  | Clinical skills |
|  |  |  |  |  |  |  | Diagnostic skills |
|  |  |  |  |  |  |  | Advisory skills |
|  |  |  |  |  |  |  | Reaction to emergency calls |
|  |  |  |  |  |  |  | Punctuality |
|  |  |  |  |  |  |  | Human relations |
|  |  |  |  |  |  |  | Provider of preventive services |
|  |  |  |  |  |  |  | Ethically and legally bounded |
|  |  |  |  |  |  |  | Model of profession |
|  |  |  |  |  |  |  | Communication of health issues |
|  |  |  |  |  |  |  | Abilities in planning and evaluation |
|  |  |  |  |  |  |  | Health policy understanding |
|  |  |  |  |  |  |  | Advocating of issues related to health |
|  |  |  |  |  |  |  | Advisor of health cultures |
|  |  |  |  |  |  |  | Relationship with colleagues |
|  |  |  |  |  |  |  | Abilities in counseling |

1. In general how is the graduate from MUHAS compared to graduates from other Universities

O Much better O better O equally good O worse off

**MUHIMBILI UNIVERSITY OF HEALTH AND ALLIED SCIENCES (MUHAS)**

# GUIDELINES FOR TRACER STUDIES FOR MUHAS GRADUATES OF 2004-2008

## QUESTIONNAIRE

**FOR THE END-USERS OF MUHAS GRADUATES**

***(Patients)***

Good morning/afternoon/evening. My name is ___________________________.

The Muhimbili University of Health and Allied Sciences (MUHAS)

in collaboration with the Ministry of Health and Social Welfare (MOHSW) of Tanzania, is doing a survey on the contribution of end users of its graduates on its academic programmes.

This survey will help the University understand and document the findings that will guide in its curriculum review process.

You as a stakeholder are requested to participate in this study responding to questions.

The main areas include a broad overview concerning the knowledge and skills, performance, responsibility, professional career development and attitude towards work of the MUHAS graduates working in this institution.

We assure you that your responses will only be used for the purpose of this survey. In the description of the findings of this survey no identification of individual persons will be made.

**YOUR INFORMATION WILL BE TREATED WITH STRICT CONFIDENTIALITY**.

We will be pleased to send you an overview of the most important results if you wish.

Do you have any questions about this survey or about your participation? Would you like to participate?

Thank your very much for your kind participation.

**Questionnaire no: |__|__|__|__|**

**Date:______________**

**A. GENERAL INFORMATION**

Region ………………………………………..

District ………………………………………

Name of the Institution …………………………………………..

Outpatient

Inpatient

Other/specify

Outpatient/ Inpatient …………………………………………..

**B. SCREEN QUESTIONS**

***Please fill in the bubbles, following this example:***

***
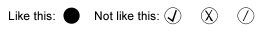
***

**Biographical Data**

1. Gender: O Male O Female
2. Age: **|__|__|** years
3. Occupation: …………………………………

**C. SURVEY SPECIFIC QUESTIONS**

1. Is this the first time you attend in this institution/health facility for care/treatment?

O Yes O No

1. For how long have you lived in this area?

**|__|__||** Months  **|__|__|** Years

1. How often have you been attending this institution/health facility for treatment/care?

*Number of visit*|**__|__|**

1. What motivates you to seek medical care/treatment in this institution/health facility?

| Excellent | | Average | | | Worse |
| --- | --- | --- | --- | --- | --- |
|  |  |  |  |  | Service Provision |
|  |  |  |  |  | Reaction to emergencies |
|  |  |  |  |  | Advisory and Counseling services |
|  |  |  |  |  | Communication services |
|  |  |  |  |  | Confidentiality |
|  |  |  |  |  |  |

**Questions 5-10 apply only to *MUHAS graduates***

1. Whom among the practitioner do you mostly consult?

………………………………………………………………

1. How do you assess the directives provided by your health practitioner?

O Very clear O clear O ambiguous

1. What is your perception toward the services rendered by this health practitioner?

O Excellent O Average O Bad

1. What motivates you to seek medical care from this health practitioner?

###### (Multiple reply possible.)

|  | |  | | |  |
| --- | --- | --- | --- | --- | --- |
|  |  |  |  |  | Willingness to listen carefully to my health problems |
|  |  |  |  |  | Willingness to attend to my problems |
|  |  |  |  |  | Ability to give clear instructions |
|  |  |  |  |  | Self-confidence of the practitioner while attending to my problems |
|  |  |  |  |  | Empathy (***Caring and being concerned without being involved***) |
|  |  |  |  |  | Ability to understand patients/customers |

1. In general how is this health practitioner compared to others?

O Much better O better O equally good O worse off

**Focus Group Discussion – Employers/ Supervisors.**

1. **In general what can you say about recent MUHAS graduates in your institution? (*To get general picture)***
2. **In your view what strengths do MUHAS graduates posses?**

**(*To assess 7 competencies*)**

**A list of the competencies should be provided herewith.**

1. **(a). What do you think are the most compelling challenges that MUHAS graduates encounter in their day to day practice?**
   1. **In your opinion do you think the training at MUHAS prepares them to respond to such challenges adequately? *(A list of possible challenges should be provided herewith)***
2. **From your assessment are there are any weaknesses in the quality of medical doctors and specialists trained at MUHAS?**

**(To assess weaknesses)**

1. **What measures would you suggest to improve the quality of medical doctors and specialists education in at MUHAS?**

**(Proposed modification)**

1. **Do you have any general opinion or comment about MUHAS, its graduates and/or this survey?**
