# Supplementary material for: The Doctor of Medicine curriculum review at the School of Medicine, Muhimbili University of Health and Allied Sciences, Dar es Salaam, Tanzania: a tracer study report from 2009
Source: BMC Med Educ. 2016 Aug 25;16(1):223. doi: 10.1186/s12909-016-0745-7 (PMC5000497; doi:10.1186/s12909-016-0745-7)
Supplement: Additional file 3: — List of General and Specific Program Competencies. (DOC 36 kb) [file 12909_2016_745_MOESM3_ESM.doc]

**Additional file 3**

**List of General and Specific Program Competencies**

**A: General Competencies**

**Relationships with Patients, Clients and Communities**

- Establish constructive relationships and communicate effectively with patients, clients and/or communities in order to address their needs and preferences
- Provide service to individuals and groups that is appropriate to their different backgrounds
- Communicate health issues and polices effectively to the public

**Relationships with Colleagues**

- Listen to and take advice from colleagues
- Motivate colleagues
- Contribute effectively to team work
- Work effectively with other health professionals

**Teaching Skills**

- Prepare and deliver effective health promotion messages to educate communities
- Teach a course for health professionals or students

**Maintaining Good Practice**

- Systematically evaluate one’s own performance and practice
- Regularly seek information necessary to improve professional practice (life-long learning)
- Apply evidence-based decision making
- Participate in applied research activities
- Use information technology to optimize learning
- Show leadership and managerial skills

**Working Within the System and Context of Health Care**

- Show knowledge of how the health care system functions (structures, policies, regulations, standards and guidelines)
- Work effectively in various health care delivery settings and systems (hospitals, government, ministries, NGO’s, communities, industry)
- Coordinate and implement health service delivery and health interventions within the health care system
- Incorporate considerations of cost effectiveness into health service delivery
- Incorporate considerations of patient cost burden into health service delivery
- Promote quality care in health systems through audits, accreditations, and/or evaluations
- Identify system challenges and implement potential solutions

**Professionalism**

- Maintain ethical standards (confidentiality, informed consent, avoid practice errors, avoid conflicts of interest)
- Apply entrepreneurial skills for advancement of practice and the profession
- Show sensitivity and responsiveness to diversity (culture, age, socioeconomic status, gender, religion, and disability)
- Show respect, compassion, and integrity while interacting with patients, clients, communities and health professionals
- Advocate and implement fair distribution of health care resources in Tanzania

**B: Specific Competencies for Doctor of Medicine (MD) Graduates**

**Professional Knowledge**

- Employ knowledge of the structure and functions of human body in management of diseases
- Employ knowledge of the causes and pathophysiology of diseases to manage diseases
- Employ knowledge of physical, psychological and socio-cultural factors in the causation and progression of diseases to plan an approach to prevent and manage common health challenges
- Employ knowledge of clinical reasoning to solve clinical problems
- Employ knowledge of pathophysiology of communicable diseases prevalent in Tanzania to diagnose and manage patients with for example HIV and AIDS, Malaria, TB, Cholera
- Employ knowledge of pathophysiology of non-communicable diseases prevalent in Tanzania to diagnose and manage patients with for example diabetes, heart diseases and cancer
- Employ knowledge of common surgical conditions prevalent in Tanzania for management of patients with for example tropical ulcer, hernia, hydrocoele
- Employ knowledge of common obstetrics and Gynaecology conditions prevalent in Tanzania for management of patients with for example prolonged labour, abruptio placenta, postpartum haemorrhage,

**Practical/ Clinical Skills**

- Gather complete and focused histories in an organized manner, appropriate to the clinical situation and patient or relative’s ability to understand
- Conduct complete and relevant physical examination in a systematic manner
- Document the findings in an organized and comprehensive manner
- Formulate and prioritize correct and appropriate plans for patient management
- Perform common procedures and alleviate patients’ pain associated with procedures
- Follow universal precautions and sterile technique
- Anticipate patients’ needs, provide appropriate patient care, participate in discharge planning and create individualized disease management and/or prevention plans including patient self-management and behaviour change
- Show confidence and comfort with the primary provider role and the provision of longitudinal care
