# Supplementary material for: The Doctor of Medicine curriculum review at the School of Medicine, Muhimbili University of Health and Allied Sciences, Dar es Salaam, Tanzania: a tracer study report from 2009
Source: BMC Med Educ. 2016 Aug 25;16(1):223. doi: 10.1186/s12909-016-0745-7 (PMC5000497; doi:10.1186/s12909-016-0745-7)
Supplement: Additional file 4: — Quantitative Tables. (DOC 234 kb) [file 12909_2016_745_MOESM4_ESM.doc]

**Additional file 4:**

Quantitative Tables

| **Table 1: MD graduates interviewed for the 2009 Tracer Study** | | | | | |
| --- | --- | --- | --- | --- | --- |
|  | | Total number who graduated | Number interviewed | Number interviewed as a % of those who graduated | Percentage interviewed by year of graduation |
| **Graduation**  **year** | 2006 | 134 | 12 | 9.0 | 8.2 |
|  | 2007 | 175 | 28 | 16 | 19.0 |
|  | 2008 | 201 | 103 | 51.2 | 70.1 |
|  | 2009 |  | 1 | - | 0.7 |
|  | Missing |  | 3 | - | 2.0 |
| **Total** |  | **510** | **147** | **29.2** | **100.0** |

| **Table 3: Demographics of the graduates interviewed** | | | | |  |
| --- | --- | --- | --- | --- | --- |
|  | | | **Number** | **%** |  |
| **Sex** | | Male | 103 | 70.1 |  |
|  |  | Female | 44 | 29.9 |  |
|  |  | Missing | 0 | 0.0 |  |
| **Total** | |  | **147** | **100.0** |  |
| **Age in years** | | 25 | 6 | 4.1 |  |
|  |  | 26 | 13 | 8.8 |  |
|  |  | 27 | 17 | 11.6 |  |
|  |  | 28 | 41 | 27.9 |  |
|  |  | 29 | 28 | 19.0 |  |
|  |  | 30 | 15 | 10.2 |  |
|  |  | 31 | 6 | 4.1 |  |
|  |  | 32 | 5 | 3.4 |  |
|  |  | 34 | 2 | 1.4 |  |
|  |  | 35 | 2 | 1.4 |  |
|  |  | 38 | 1 | .7 |  |
|  | | 39 | 3 | 2.0 |  |
|  | | Missing | 8 | 5.4 |  |
| **Total** | |  | **147** | **100.0** |  |
| **Employment status** | | Intern | 100 | 68.0 | |
|  |  | Full time | 33 | 22.4 | |
|  |  | Part time | 1 | .7 | |
|  |  | Contract | 13 | 8.8 | |
|  |  | Missing | 0 | 0.0 | |
| **Total** | |  | **147** | **100.0** | |
| **Employing organization** | Ministry or district health administration | | 37 | 25.2 | |
|  | National hospital | | 14 | 9.5 | |
|  | Regional or referral hospital | | 34 | 23.1 | |
|  | Private or religious hospital | | 11 | 7.5 | |
|  | University or training institution | | 9 | 6.1 | |
|  | International non-governmental organization or agency | | 1 | .7 | |
|  | Other | | 17 | 11.6 | |
|  | Missing | | 24 | 16.3 | |
| **Total** |  | | **147** | **100.0** | |

| **Table 4a: Competencies common to all MUHAS graduates** | | | |
| --- | --- | --- | --- |
| **Competence**  Figures in cells are the percentages agreeing, mean (SD) of Likert Scale from 1-5, where 1= strongly disagree and 5= strongly agree | Graduate  n=147  I was “trained to” | Supervisor  n=48  “Professionals are expected to” | Supervisor  n=48  “Graduate is able to” |
| B1: Relationships with patients |  |  |  |
| Establish constructive relationships and communicate effectively with patients, clients and/or communities in order to address their needs and preferences | 84.9%  4.29 (0.90) | 95.6%  4.71 (0.73) | 80.0%  3.98 (0.89) |
| Provide service to individuals and groups that is appropriate to their different backgrounds | 80.6%  4.17(1.02) | 95.5%  4.70 (0.73) | 72.7%  3.82 (1.10) |
| Communicate health issues and polices effectively to the public | 69.2%  3.92 (1.08) | 95.3%  4.74 (0.73) | 72.1%  3.79 (1.08) |
| **B2: Relationships with colleagues** |  |  |  |
| Listen to and take advice from colleagues | 84.2%  4.34 (1.06) | 97.7%  4.82 (0.66) | 68.3%  3.68 (1.16) |
| Motivate colleagues | 72.6%  3.97 (1.11) | 97.6%  4.68 (0.72) | 56.8%  3.55 (1.13) |
| Contribute effectively to team work | 82.2%  4.27 0(.90) | 97.7%  4.88 (0.62) | 65.9%  3.86 (0.95) |
| Work effectively with other health professionals | 84.7%  4.30 (1.00) | 97.7%  4.82 (0.66) | 75.0%  4.07 (.90) |
| **B3: Teaching** |  |  |  |
| Prepare and deliver effective health promotion messages to educate communities | 67.8%  3.84 (1.08) | 95.3%  4.58 (0.76) | 52.3%  3.50 (1.02) |
| Teach a course for health professionals or students | 65.3%  3.78 (1.26) | 86.4%  4.50 (0.93) | 46.5%  3.51 (0.93) |
| **B4: Good practice** |  |  |  |
| Systematically evaluate one’s own performance and practice | 64.8%  3.69 (1.11) | 95.5%  4.57 (0.76) | 34.1%  3.27 (1.06) |
| Regularly seek information necessary to improve professional practice (life-long learning) | 75.3%  4.05 (1.10) | 97.7%  4.77 (0.68) | 59.1%  3.66 (1.14) |
| Apply evidence-based decision making | 74.3%  4.03 (1.04) | 97.6%  4.67 (0.72) | 59.5%  3.57 (1.00) |
| Participate in applied research activities | 67.8%  3.88 (1.16) | 90.2%  4.51 (0.75) | 50.0%  3.41 (1.23) |
| Use information technology to optimize learning | 50.7%  3.48 (1.20) | 97.6%  4.71 (0.60) | 55.0%  3.65 (1.08) |
| Show leadership and managerial skills | 56.6%  3.66 (1.15) | 95.3%  4.65 (0.65) | 47.7%  3.30 (1.17) |
| **B5: Health care systems** |  |  |  |
| Show knowledge of how the health care system functions (structures, policies, regulations, standards and guidelines) | 60.0%  3.80 (0.94) | 90.7%  4.56 (0.73) | 44.4%  3.44 (1.08) |
| Work effectively in various health care delivery settings and systems (hospitals, government, ministries, NGO’s, communities, industry) | 63.2%  3.83 (1.00) | 97.7 %  4.72 (0.59) | 64.4%  3.82 (1.01) |
| Coordinate and implement health service delivery and health interventions within the health care system | 66.9%  3.83 (1.048) | 97.7%  4.60 (0.62) | 53.3v  3.47 (0.94) |
| Incorporate considerations of cost effectiveness into health service delivery | 63.4%  3.83 (1.02) | 88.4%  4.51 (0.77) | 52.3%  3.45 (1.00) |
| Incorporate considerations of patient cost burden into health service delivery. | 67.6%  3.83 (1.04) | 90.9%  4.50 (0.73) | 50.0%  3.39 (1.06) |
| Promote quality care in health systems through audits, accreditations, and/or evaluations | 48.3%  3.43 (1.22) | 93.0%  4.56 (0.70) | 46.5%  3.33 (1.15) |
| Identify system challenges and implement potential solutions | 55.2%  3.59 (1.18) | 94.9%  4.56 (0.79) | 46.5%  3.44 (1.16) |
|  |  |  |  |
| Maintain ethical standards (confidentiality, informed consent, avoid practice errors, avoid conflicts of interest) | 94.5%  4.59 (.70) | 97.7%  4.77 (0.68) | 73.9%  3.87 (1.24) |
| Apply entrepreneurial skills for advancement of practice and the profession | 51.0%  3.46 (1.28) | 88.1%  4.38 (0.901) | 51.1%  3.47 (1.16) |
| Show sensitivity and responsiveness to diversity (culture, age, socioeconomic status, gender, religion, and disability) | 70.3%  4.03 (1.03) | 97.7%  4.65 (.72) | 63.6%  3.73 (1.17) |
| Show respect, compassion, and integrity while interacting with patients, clients, communities and health professionals | 88.3%  4.44 (0.86) | 97.7%  4.74 (0.69) | 68.9%  3.80 (1.14) |
| Advocate and implement fair distribution of health care resources in Tanzania | 64.3%  3.85 (1.14) | 90.7%  4.63 (0.72) | 51.2%  3.47 (1.12) |

| **Table 4b: Competencies related to professional knowledge of MD graduates** | | | |
| --- | --- | --- | --- |
| **Competence**  Figures in cells are the percentages agreeing, mean (SD) of Likert Scale from 1-5, where 1= strongly disagree and 5= strongly agree | Graduate  n=147  I was “trained to” | Supervisor  n=48  “Professionals are expected to” | Supervisor  n=48  “Graduate is able to” |
| **C1: Professional Knowledge** |  |  |  |
| Employ knowledge of the structure and functions of human body in management of diseases | 90.3%  4.45 (0.76) | 97.7%  4.79 (0.67) | 82.2%  4.09 (.973) |
| Employ knowledge of causes and mechanism of diseases to manage them | 92.4%  4.46 (.71) | 97.6%  4.76 (0.69) | 75.6%  3.89 (1.07) |
| Employ knowledge of physical, psychological and socio-culture factors in the causation and progression of diseases to plan an approach to prevent and manage common health challenges | 87.4%  4.31 (0.86) | 97.7%  4.86 (0.63) | 61.4%  3.82 (0.92) |
| Employ knowledge of clinical reasoning to solve clinical problems | 90.9%  4.49 (0.73) | 97.7%  4.84 (0.65) | 80.0%  4.04 (0.88) |
| Employ knowledge of pathophysiology of communicable diseases prevalent in Tanzania to diagnose and manage patients with for example HIV and AID, Malaria, TB, Cholera | 92.3%  4.49 (0.74) | 95.2%  4.76 (0.73) | 81.8%  4.30 (0.82) |
| Employ knowledge of pathophysiology of non-communicable diseases prevalent in Tanzania to diagnose and manage patients with for example diabetes, heart diseases and cancer | 87.3%  4.30 (0.82) | 97.4%  4.82 (0.68) | 74.4%  3.98 (0.83) |
| Employ knowledge of common surgical conditions prevalent in Tanzania for management of patients with for example tropical ulcer, hernia, hydrocoele | 71.1%  4.03 (1.08) | 97.3%  4.78 (0.71) | 70.7%  3.68 (1.21) |
| Employ knowledge of common obstetrics and Gynaecology conditions prevalent in Tanzania for management of patients with for example prolonged labour, abruptio placenta, postpartum haemorrhage | 90.3%  4.53 (0.83) | 94.4%  4.69 (0.79) | 75.0%  3.83 (1.13) |

| **Table 4c: Competencies related to practical/clinical skills of MD graduates** | | | |
| --- | --- | --- | --- |
| **Competence**  Figures in cells are the percentages agreeing, mean (SD) of Likert Scale from 1-5, where 1= strongly disagree and 5= strongly agree | Graduate  n=147  I was “trained to” | Supervisor  n=48  “Professionals are expected to” | Supervisor  n=48  “Graduate is able to” |
| **C2: Practical/Clinical Skills** |  |  |  |
| Gather complete and focused histories in an organized manner, appropriate to the clinical situation and patient or relative's ability to understand | 87.5%  4.38 (0.83) | 97.7%  4.74 (0.69) | 65.9%  3.68 (1.07) |
| Conduct complete and relevant physical examination in a systematic manner | 92.4%  4.51 (0.87) | 97.7%  4.86 (0.64) | 65.9%  3.91 (0.98) |
| Document the findings in an organized and comprehensive manner | 91.0%  4.43 (.87) | 95.2%  4.71 (074) | 50.0%  3.61 (1.06) |
| Formulate and prioritize correct and appropriate plans for patient management | 87.5%  4.42 (0.91) | 95.3%  4.77 (0.72) | 62.2%  3.67 (0.93) |
| perform common procedures and alleviate patient's pain associated with procedures | 68.5%  3.91 (1.14) | 95.3%  4.77 (0.72) | 62.2%  3.71 (1.25) |
| Follow universal precautions and sterile technique | 81.4%  4.14 (.990 | 97.5%  4.85 (0.70) | 76.3%  4.17 (0.85) |
| Anticipate patient's need, provide appropriate patient care, participate in discharge planning and create individualized disease management and/or prevention plans including patient self-management and behavior change | 76.6%  4.06 (0.99) | 97.6%  4.73 (0.71) | 53.5%  3.63 (1.15) |
| Show confidence and comfort with the primary provider role and the provision of longitudinal care | 69.9%  3.97(1.10) | 97.5%  4.68 (0.73) | 62.5%  3.67 (0.92) |

| **Table 5: Graduates opinions of courses undertaken at MUHAS** | | | | |
| --- | --- | --- | --- | --- |
| Figures in cells are the means of Likert Scale from 1-5, where 1= strongly disagree and 5= strongly agree | This course prepared me for my current professional needs  n=147 | | MUHAS prepared me to take this course  n=147 | |
| Figures in cells are based on a Likert Scale from 1-5, where 1= strongly disagree and 5= strongly agree | Percentage  agree | Mean (SD) | Percentage  agree | Mean (SD) |
| Anatomy | 70.9% | 3.96 (1.11) |  |  |
| Biochemistry | 56.5% | 3.59 (1.18) |  |  |
| Medical Ethics I | 80.0% | 4.11 (1.05) |  |  |
| Physiology | 83.6% | 4.30 (0.90) | 75.9% | 4.04 (1.13) |
| Behavioral Sciences | 65.0% | 3.82 (1.04) | 56.5% | 3.50 (1.33) |
| Development studies | 60.0% | 3.69 (1.15) | 50.0% | 3.38 (1.35) |
| Microbiology/Immunology | 79.3% | 4.21 (0.96) | 74.8% | 3.97 (1.06) |
| Parasitology/Medical Entomology | 80.0% | 4.19 (0.94) | 79.0% | 4.03 (1.06) |
| Clinical Physiology | 82.0% | 4.24 (0.97) | 78.8% | 4.16 (1.04) |
| Development studies | 61.0% | 3.64 (1.13) | 59.0% | 3.61 (1.28) |
| Introduction to Clinical Medicine | 67.6% | 3.83 (1.24) | 67.8% | 3.84 (1.32) |
| Pathology | 70.8% | 4.02 (1.05) | 73.3% | 3.99 (1.17) |
| Epidemiology and Research Methods | 81.2% | 4.17 (0.92) | 63.6% | 3.76 (0.11) |
| Nutrition Field Project | 71.3% | 3.95 (1.14) | 72.0% | 3.95 (1.12) |
| Introduction to Clinical Medicine | 67.4% | 3.84 (1.27) | 71.4% | 3.92 (1.24) |
| Forensic Pathology | 39.9% | 2.91 (1.45) | 45.4% | 3.13 (1.46) |
| Clinical Pharmacology | 73.5% | 3.96 (1.12) | 70.1% | 3.86 (1.22) |
| Management of Disease I | 75.7% | 4.04 (1.12) | 79.8% | 4.08 (1.09) |
| Medical Ethics II | 75.0% | 4.06 (1.09) | 70.0% | 3.88 (1.20) |
| Medical Ethics III | 76.6% | 4.10 (1.04) | 71.7% | 3.96 (1.14) |
| Management of Disease II | 79.6% | 4.19 (1.00) | 71.7% | 4.24 (1.00) |
| Community medicine | 74.1% | 4.00 (1.12) | 76.1% | 4.01 (1.11) |
| Paediatrics & Child Health | 82.0% | 4.20 (1.08) | 77.8% | 4.13 (1.10) |
| Obstetrics & Gynaecology | 92.1% | 4.50 (0.78) | 85.3% | 4.36 (0.94) |
| Elective Period | 72.8% | 4.05 (1.01) | 76.4% | 4.07 (1.08) |
| Surgery | 82.0% | 4.21 (0.97) | 80.2% | 4.15 (1.01) |
| Internal medicine | 85.4% | 4.35 (0.98) | 80.0% | 4.19 (1.07) |
| Surgical Specialties | 77.5% | 4.12 (1.04) | 76.3% | 4.14 (1.10) |
| Orthopaedics & Traumatology | 64.5% | 3.78 (1.25) | 66.4% | 3.82 (1.28) |
| Psychiatry | 86.8% | 4.45 (0.81) | 81.1% | 4.21 (1.00) |

| **Table 6: Graduates opinions of the learning/teaching environment (**n=147) | | | |
| --- | --- | --- | --- |
| Figures in cells are based on a Likert Scale from 1-5, where 1= strongly disagree and 5= strongly agree | | Percentage  agree | Mean (SD) |
| “How useful were the following teaching method to helping you learn?” | Lectures | 79.9% | 4.07 (0.87) |
|  | Seminars/Tutorials | 69.9% | 3.90 (1.02) |
|  | Laboratory skills/Practicals | 51.7% | 3.54 (1.11) |
|  | Theatre skills | 45.5% | 3.39 (1.33) |
|  | Teaching ward rounds | 80.6% | 4.12(0.99) |
|  | Outpatients clinics | 66.2% | 3.80 (1.02) |
|  | Family case studies | 47.2% | 3.37 (1.19) |
|  | Elective studies | 75.0% | 4.04 (0.91) |
|  | Field work | 78.6% | 4.17 (0.92) |
|  | Presentation | 86.8% | 4.32 (0.82) |
|  | Self reflection | 68.3% | 3.89 (1.03) |
|  | visits/excursions | 31.7% | 3.39 (1.58) |
| “The following meet my learning needs” | Computer lab | 21.5% | 2.27 (1.35) |
|  | Libraries | 60.1% | 3.46 (1.23) |
|  | Internet Access | 23.9% | 2.39 (1.37) |
| “How useful were the following assessment methods to your learning?” | Continuous assessments | 82.2% | 4.17 (0.92) |
|  | Final examinations | 76.7% | 4.14 (0.91) |
|  | Multiple choice | 72.1% | 3.88 (0.93) |
|  | Essays | 76.0% | 3.96 (0.95) |
|  | Short answer s | 80.4% | 4.06 (0.87) |
|  | Oral examination | 68.0% | 3.81 (1.23) |
|  | Field work/projects | 72.0% | 4.16 (0.90) |
|  | Clinical/practical examination | 84.8% | 4.28 (0.88) |
|  | Clinical logbooks | 40.2% | 3.24 (1.37) |
|  | Research report | 70.5% | 3.88 (0.99) |
|  | Presentations | 81.8% | 4.22 (0.94) |
| “Rate MUHAS teachers in the following roles” | Professional role modeling by teachers | 47.9% | 3.24 (1.13) |
|  | Accessibility of teachers outside scheduled teaching sessions | 19.0% | 2.34 (1.15) |
|  | Monitoring of students | 31.5% | 2.77 (1.20) |
|  | Teaching sessions | 57.9% | 3.51 (1.02) |
|  | Academic advice given | 31.7% | 2.83 (1.28) |
|  | Nature of your relationship with teaching staff | 23.3% | 2.68 (1.25) |

**^+^Scale range:** *1-5, where 1= strongly disagree and 5= strongly agree*
