# Supplementary material for: The Doctor of Medicine curriculum review at the School of Medicine, Muhimbili University of Health and Allied Sciences, Dar es Salaam, Tanzania: a tracer study report from 2009
Source: BMC Med Educ. 2016 Aug 25;16(1):223. doi: 10.1186/s12909-016-0745-7 (PMC5000497; doi:10.1186/s12909-016-0745-7)
Supplement: Additional file 5: — MD Programme Summaries. (DOC 109 kb) [file 12909_2016_745_MOESM5_ESM.doc]

**Additional** **file 5:**

**Appendix E**

**Summary of the existing MD program courses/structure under review during the study**

| **Course** | **Lectures** | **Practical/Seminars** | **Total Hours** | **Teaching weeks** | **Units** |
| --- | --- | --- | --- | --- | --- |
| **SEMESTER 1 - 25.6 UNITS** | | | | | |
| AN 100 | 110 | 232 | 342 | 19 | 13.8 |
| BC 100 | 148 | 41 | 189 | 19 | 10.7 |
| MF 301 | 17 | - | 19 | 19 | 1.1 |
| **SEMESTER 2 - 24.2 UNITS** | | | | | |
| PH 100 | 113 | 74 | 187 | 19 | 9.2 |
| BS 100 | 129 | 80 | 209 | 19 | 10.4 |
| DS 100 | 60 | 30 | 90 | 19 | 4.6 |
| **SEMESTER 3 - 26.3 UNITS** | | | | | |
| MM 200 | 44 | 76 | 190 | 19 | 9.4 |
| PE 200 | 91 | 61 | 152 | 19 | 7.3 |
| PH 200 | 57 | - | 57 | 19 | 3.7 |
| DS 200 | 60 | 30 | 90 | 19 | 4.9 |
| CM 200 | - | 45 | 45 | 19 | 1.0 |
| **SEMESTER 4 - 32.8 UNITS** | | | | | |
| MP 200 | 199 | 68 | 267 | 19 | 14.7 |
| ER 200 | 65 | 167 | 232 | 19 | 8.0 |
| EF 200 | 50 | 90 | 140 | 19 | 5.3 |
| CM 200 | 37 | 45 | 82 | 19 | 3.5 |
| MF 302 | 19 | 8 | 19 | 19 | 1.3 |
| **SEMESTER 5 - 31.1 UNITS** | | | | | |
| ML 300 | 164 | 26 | 190 | 19 | 11.4 |
| MD 300 (301-309) | 264 | 53 | 334 | 19 | 18.8 |
| MF 303 | 11 | - | 19 | 19 | 0.9 |
| SEMESTER 6 - 33.7 UNITS | | | | | |
| MF 304 | 17 | - | 19 | 19 | 1.1 |
| MD 300 (310-318) | 422 | 167 | 589 | 19 | 32.6 |
| **SEMESTER 7and 8 - 33.3 UNITS** | | | | | |
| MC 400 | 15 | 369 | 15 | 12 | 9.2 |
| MH 400 | 15 | 369 | 15 | 12 | 9.2 |
| MG 400 | 15 | 369 | 15 | 12 | 9.2 |
| ME 400 |  | 257 | 256 | 8 | 5.7 |
| **SEMESTER 9 and 10 - 34.6 UNITS** | | | | | |
| MS 500 | 15 | 369 | 384 | 12 | 10.7 |
| MI 500 | 15 | 369 | 384 | 12 | 9.8 |
| MZ 500 | 48 | 144 | 192 | 6 | 6.4 |
| MY 500 | 48 | 144 | 192 | 6 | 6.4 |

**Key**

AN 100: Anatomy

BC 100: Biochemitry

MF 301: Medical Ethics I

PH 100: Basic Physiology

BS 100: Behavioural Science

DS 100: Development studies 100

DS 200: Development studies 200

MM 200: Microbiology and Immunology

PE 200: Parasitology and Entomology

PH 200: Clinical Physiology

CM 200: Introduction to Clinical Medicine

MP 200: Pathology

MF 302: Forensic Pathology

ER 200: Epidemiology and Research Methodology

EF 200: Nutrition Field Project

ML 300: Clinical Pharmacology

MF 304: Medical Ethics II

MD 300: Management of Diseases

MC 400: Community Medicine

MH 400: Paediatrics and Child Health

MG 400: Obstetrics and Gynaecology

ME 400: Elective period

MS 500: Surgery

MI 500: Internal Medicine

MZ 500: Surgical specialities

MY 500: Psychiatry

**Appendix F**:

|  | **PROPOSED MODULARIZED MD CURRICULUM JULY 8^TH^ 2010** | | | | | | | | | | | |
| --- | --- | --- | --- | --- | --- | --- | --- | --- | --- | --- | --- | --- |
|  | **Biomedical Sciences, 72 Weeks** | | | |  | **Junior Clerkship, 40 Weeks** | **Senior Clerkship, 80 Weeks** | | | | | |
|  | **YEAR 1** | | **YEAR 2** | |  | **YEAR 3** | **YEAR 4** | | | | **YEAR 5** | |
|  | **Semester 1** | **Semester 2** | **Semester 3** | **Semester 4** |  | **(Semester 5-6)** | **(Semester 7-8)** | | | | **(Semester 9-10)** | |
|  | *18 wks x32hs = 576 hrs* | *18 wks x32hs = 576 hrs* | *18 wks x32hs = 576 hrs* | *18 wks x32hs = 576 hrs* |  | *40 Weeks* | *40 Weeks* | | | | *40 Weeks* | |
|  | Anatomy I | Anatomy II | Microbiology | Psychopathology |  | Internal Medicine | | **9** | Psychiatry | **8** | Internal Medicine | **8** |
| **Hours** | **140** | **200** | **190** | **36** |  |  |  |  |  |  |  |  |
|  | Biochemistry I | Biochemistry II | Epidemiology/Res Methods I, (Family Case Study, Env and Occ Health) | Epid/Res Methodology II, |  | Pediatrics/Child Health | | **9** | Community Medicine | **8** | Pediatrics/Child Health | **8** |
| **Hours** | **100** | **110** | **90** | **90** |  |  |  |  |  |  |  |  |
|  | Physiology I | Physiology II | Pathology I, (includes MF) | Pathology II |  | Surgery | | **9** | ENT | **8** | Surgery | **8** |
| **Hours** | **150** | **120** | **116** | **170** |  |  |  |  | Ophthalmology | **8** |  |  |
|  | Medical Ethics | Medical Ethics | Parasitology/Entomology |  |  | Obstetrics/Gynecology | | **9** | Anaesthesiology | **8** | Obstetrics/Gynecology | **8** |
|  |  |  |  |  |  | Medical Ethics | |  |  |  | Orthopedics/Trauma | **8** |
|  |  |  |  |  |  | **36 hours** | |  |  |  |  |  |
| **Hours** | **36** | **36** | **110** |  |  | CDC (Communicable Disease Control) | |  |  |  |  |  |
|  |  |  |  |  |  | **144 hours** | |  |  |  |  |  |
|  |  |  |  | Clinical Pharmacology |  | Radiology | |  |  |  |  |  |
|  |  |  |  |  |  | **72 hours** | |  |  |  |  |  |
| **Hours** |  |  |  | **180** |  | **Total is 4 Weeks, before rotations** | | **4** |  |  |  |  |
|  |  |  |  |  |  |  |  |  |  |  |  |  |
|  |  | DS 100 | DS 200 | Nutr. Field Project Lectures |  |  | |  |  |  |  |  |
| **Hours** |  | **70** | **70** | **20** |  |  | |  |  |  |  |  |
|  | BS 100 |  |  |  |  |  | |  |  |  |  |  |
| **Hours** | **150** |  |  |  |  |  | |  |  |  |  |  |
| **Total Hrs** | **576** | **536** | **576** | **496** |  | ***Total weeks*** | | **40** |  | **40** |  | **40** |
| **Deficit** | **0** | **40** | **0** | **80** |  |  | |  |  |  |  |  |
|  |  |  |  |  |  |  | |  |  |  |  |  |
| **Elective Period** | | To be done after semester 8 | | |  |  | |  |  |  |  |  |
|  |  | To be co-ordinated by School of Medicine | | | |  | |  |  |  |  |  |
|  |  |  |  |  |  |  | |  |  |  |  |  |
| **Nutrition Field Project** | | To be done after semester 3 | | |  |  | |  |  |  |  |  |
|  |  | To be co-ordinated by Community Health | | | |  | |  |  |  |  |  |
